# Association of collective attitudes and contraceptive practice in nine sub-Saharan African countries

**DOI:** 10.7189/jogh.10.010705

**Published:** 2020-06

**Authors:** Iván Mejía-Guevara, Beniamino Cislaghi, Ann Weber, Emma Hallgren, Valerie Meausoone, Mark R Cullen, Gary L Darmstadt

**Affiliations:** 1 Center for Population Health Sciences, Stanford University School of Medicine, Palo Alto, California, USA; 2 Department of Biology, Stanford University, Palo Alto, California, USA; 3 London School of Hygiene & Tropical Medicine, London, United Kingdom; 4 School of Community Health Sciences, University of Nevada, Reno, Reno, Nevada, USA; 5 Department of Pediatrics, Stanford University School of Medicine, Stanford, California, USA

## Abstract

**Background:**

There is ample evidence that gender norms affect contraceptive practice; however, data are mostly qualitative with limited geographical scope. We investigated that association quantitatively using collective community-level attitudes towards premarital sex and wife-beating as proxies for gender norms.

**Methods:**

Data came from nationally representative Demographic and Health Surveys (2005-2009) for women of reproductive age (15-49 years) in nine sub-Saharan African countries. Using multilevel logistic models, controlling for individual covariates and community-level indicators of women’s empowerment, we assessed the community-level association of gender norms regarding premarital sex and wife-beating with individual contraception uptake and demand satisfied among fecund sexually active women. Norms were approximated as ‘collective attitudinal norms’ from female/male residents (aged 15-49 years) from the same community. We assessed the magnitude and significance of the community-level effects and attributed variance across communities. The same analysis was replicated for each country.

**Results:**

In a fully-adjusted model with a pooled sample of 24 404 adolescent women, the odds of contraception use increased with a 1 standard deviation (SD) increase in the variation of collective permissive attitudes towards premarital sex of female (odds ratio (OR) = 1.08, 95% confidence interval (CI) = 1.02-1.15) and male (OR = 1.11, 95% CI = 1.05-1.17) peers (15-24 years), while odds of contraceptive use declined by 10% (OR = 0.90, 95% CI = 0.85-0.96) with collective accepting attitudes towards wife-beating of women aged 15-49 years. Similar results were found in separate models that controlled for adults’ permissive attitudes towards premarital sex. The community-level attributed variance (V_2_ = 1.62, 95% CI = 1.45-1.80) represented 33% (intra-class correlation (ICC) = 33.0, 95% CI = 30.0-35.4) of the total variation of contraception use, and attitudes towards premarital sex and violence jointly explained nearly 26% of that V_2_ variance. The community-level shared of attributed variation of contraceptive use varied significantly across countries, from 3.5% in Swaziland (ICC = 3.5, 95% CI = 0.8-13.7) to 60.2% in Nigeria (OR = 60.2, 95% CI = 56.0-64.2).

**Conclusions:**

Overall, significant positive associations of collective permissive attitudes of both adolescent and adult women towards premarital sex were found for use of, and demand for, contraception, whereas collective accepting attitudes towards wife-beating were negatively associated with the use and demand for contraception. Ours is the first study to define quantitatively the influence of proxies for gender norms at the community level on women’s family planning decisions. These findings offer new insights for understanding the role of sex-related attitudes and norms as important factors in shaping contraceptive practices and improving the effectiveness of family planning policies by targeting individuals as well as their groups of influence.

With a delayed fertility transition – where the total fertility rate remains high and declines slowly [1,2] – the population in sub-Saharan Africa (SSA) is growing at a faster rate than in other developing regions [3]. These fertility and population trends pose important challenges for development in the world’s poorest region [4]. Voluntary family planning programmes have been regarded as the preferred policy response to curb rapid population growth and are aimed for women and men to take control, free from coercion, of their reproductive health to avoid unintended pregnancies [5]. Further, the right of women and girls to choose whether, when and how many children to have has been recognised in international agreements and the Global Agenda for Sustainable Development [6-8].

Although important gains have been achieved in reducing unmet need for family planning, increasing contraceptive prevalence, and preventing unintended pregnancies [7,9-12], those gains have been slow [13,14], and important obstacles persist in SSA. Recent studies indicate that low socioeconomic status; financial barriers; limitations in access to information, services and supplies; and cultural norms and societal pressure on women to bear children, represent the major barriers for family planning and contraceptive uptake within the region [15]. The most common cultural norms and social obstacles cited in the literature include: misinformation regarding the use and effectiveness of contraception, fear of side effects, infrequent sex and perceptions that contraception is not needed, traditional views of women’s main role as bearers of children, parents avoiding conversations about reproductive health matters with their children, prohibition of contraceptive usage by partners or family members, fear of verbal/physical violence, and the desire for large families [16-22]. In addition, despite evidence (mostly qualitative) that norms affect family planning and contraceptive use [23-26], their examination in global data sets is challenging, as little data specifically on norms are available, with most advanced studies resorting to using proxies [27]. We have recently advanced the quantitative analysis of associations between gender norm proxies and health, constructing reference groups and deriving insights into potential influences of the normative environment on health-related behaviours [27].

This study rests on recent theoretical frameworks of normative influence [28,29] to investigate the extent to which reproductive behavior can be shaped by social relations and contextual factors. The theory suggests that social norms – beliefs about what other people from a given group or society do and approve of – affect individuals’ behaviors. Here we are particularly interested in the role of gender-related norms. Gender norms are social norms defining acceptable and appropriate actions for women and men in a given group or society. They are embedded in formal and informal institutions, nested in the mind, and produced and reproduced through social interaction. They play a role in shaping women and men's (often unequal) access to resources and freedoms, thus affecting their voice, power, and sense of self. We use the theory of normative spectrum (TNS) as an interpretive framework. The TNS suggests that a norm has a stronger influence on practice the more the practice is: 1) interdependent (vs independent), 2) detectable (vs private), 3) followed by strong (vs weak) rewards or punishments (sanctions), and 4) closely related to the norm (as opposed to distant from it). In our study, we were interested in understanding how gender norms, as reflected by two relatively undetectable practices: premarital sex and domestic violence against women, affected contraceptive use practice. We approximated these two different gender norms exposures as “collective attitudinal norms” [30-32] using summations of individual attitudes of female/male residents (aged 15-49 years) in the same community towards premarital sex and wife-beating. In the case of attitudes towards premarital sex, the measurement was based on individual attitudes of female or male peers (aged 15-24 years) or adults (aged 25-49 years) separately, following previous evidence from independent studies assessing the effect of peers or adults (parents, mothers, tutors) on adolescent decision making and family planning [33-35]. We hypothesised that women who engage in premarital sex or who don’t accept domestic violence, and are living in communities where gender norms are the reverse (against premarital sex or accepting of wife-beating), would be more reluctant to talk to others (peers, adults) about family planning matters or to use reproductive health services [27,33,34]. Our community-level variables, permissive attitudes towards premarital sex and acceptance of wife-beating, were considered as proxies for gender norms because they may reflect women’s lower sense of entitlement and self-esteem and their lack of control of their own reproductive behavior [36-38]. We acknowledge that the aggregation of individual attitudes is not equivalent to gender norms, but it provides valuable information about the general beliefs of the community.

With data from Demographic and Health Surveys (DHS), we defined individual permissive attitudes towards premarital sex by reverse-coding the question ‘should young women wait for sex until marriage?’. We approximated collective permissive attitudes towards acceptance of premarital sex from aggregated attitudes of adolescent peers (aged 15-24 years) or adults (aged 25-49 years) at the community level. Similarly, we used aggregated individual attitudes justifying wife-beating if a woman refuses to have sex with her husband to approximate collective attitudes towards wife-beating for women aged 15-49 years from the same community. We then explored the community-level association of collective attitudes towards premarital sex and wife-beating with the uptake and demand satisfied for contraception among women of reproductive age (aged 15-49 years). This study aimed to offer insights into understanding the role of gender norms, or the social norms regulating acceptable actions for men and women [39], as important barriers to contraceptive uptake in low-resource settings and to inform the design of effective interventions that target individuals as well as their groups of influence.

## METHODS

### Study design and participants

Data for this study came from DHS surveys from nine SSA countries (Benin, Congo, Mali, Namibia, Niger, Nigeria, Sao Tome and Principe, Swaziland, and Zambia) with information on attitudes towards sex before marriage for men and women from 2005 to 2009. DHS surveys are based on a stratified two-stage clustered survey design. Strata are defined by rural and urban areas and administrative units (provinces or states). In the first stage, primary sampling units (PSUs) are selected in each stratum with probability proportional to a measure of size from a sampling frame defined by a list of geographic units that covered the entire country. In selected PSUs or clusters, trained field staff conduct a listing of households that serve as a sampling frame for the second stage selection. A sample of households are then selected systematically from that listing to identify eligible women of reproductive age (aged 15-49), and face-to-face interviews are conducted with eligible women after full consent is granted. Data collected from these interviews include indicators of family planning and fertility practices/preferences, among other maternal and child health indicators [40].

**Figure 1:**
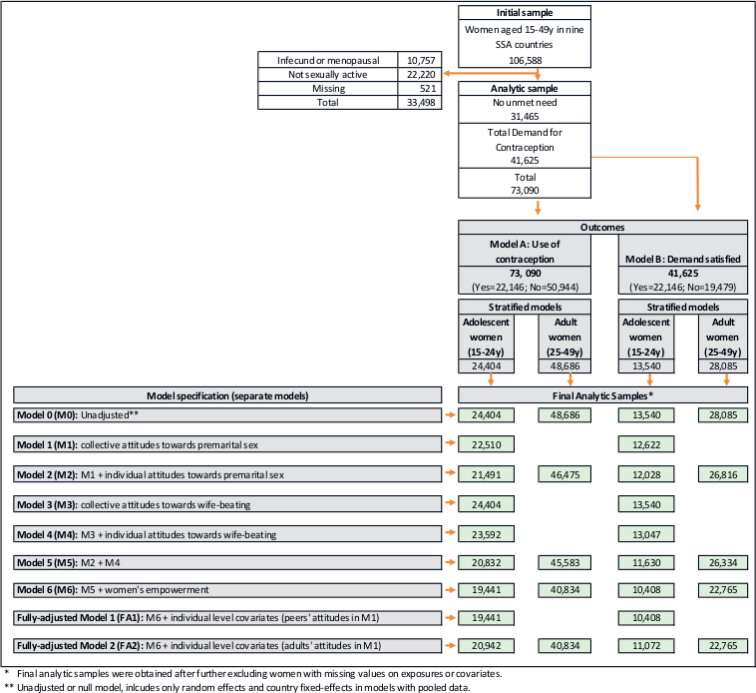
Model specification and sample size breakdown for each model. SSA – sub-Saharan Africa.

The original sample for this study was comprised of 106 588 women of reproductive age (15-49 years) that was used for descriptive analysis. Analysis of contraception use was conducted on a reduced sample of 73 090 women, after the exclusion of 10 757 (10.1%) infecund or menopausal women, 22 220 (20.8%) women not sexually active, and 521 (0.5%) women with missing information on fertility preferences. A further exclusion of 31 465 (29.5%) women with no reported unmet need for contraception resulted in a sample of 41 625 (39.1%) fecund women for the analysis of demand satisfied for contraception (**Table 1** and **Figure 1**).

**Table 1:** Sample distribution of family planning and fertility preferences of women aged 15-49 y, and community-level exposures of female/male peers (aged 15-24) and adults (aged 25-49) by age group and country

	Benin	Congo	Mali	Namibia	Niger	Nigeria	Sao Tome and Principe	Swaziland	Zambia	Pooled sample
**Family planning and fertility preferences, N (weighted %)**										
**Youth women aged 15-24 years:**										
Total demand	1987 (32.5)	1888 (60.9)	1619 (28.1)	1969 (47.1)	654 (17.8)	2874 (24.2)	447 (44.3)	970 (42.1)	1132 (37.0)	13540 (32.8)
Unmet need	1032 (16.4)	444 (14.8)	1213 (21.5)	439 (9.5)	409 (11.0)	1568 (12.1)	202 (21.7)	328 (14.3)	491 (16.2)	6126 (14.6)
Use of contraception	955 (25.7)	1444 (47.0)	406 (23.3)	1530 (10.4)	245 (15.1)	1306 (16.2)	245 (22.8)	642 (15.5)	641 (22.1)	7414 (20.8)
No unmet need	1697 (27.1)	366 (13.5)	2266 (39.4)	227 (5.0)	1452 (51.7)	4111 (30.3)	110 (10.2)	157 (6.8)	478 (16.0)	10864 (26.3)
Other	2469 (40.5)	770 (25.6)	1886 (32.6)	1885 (48.0)	1405 (30.5)	5709 (45.5)	430 (45.6)	1165 (51.1)	1393 (47.1)	17112 (40.9)
Total	6153 (100.0)	3024 (100.0)	5771 (100.0)	4081 (100.0)	3511 (100.0)	12694 (100.0)	987 (100.0)	2292 (100.0)	3003 (100.0)	41516 (100.0)
**Adult women aged 25-49 years:**										
Total demand	4907 (42.9)	2366 (57.4)	2941 (33.5)	3825 (67.6)	1803 (27.6)	6958 (36.0)	1070 (67.4)	1771 (65.2)	2444 (59.6)	28085 (43.7)
Unmet need	2931 (25.1)	598 (14.9)	2184 (25.4)	880 (14.6)	981 (15.8)	3861 (18.6)	481 (31.5)	492 (18.5)	945 (23.3)	13353 (20.4)
Use of contraception	1976 (17.8)	1768 (42.6)	757 (8.1)	2945 (53.1)	822 (11.8)	3097 (17.5)	589 (35.9)	1279 (46.7)	1499 (36.4)	14732 (23.3)
No unmet need	4153 (35.2)	891 (23.5)	3459 (40.4)	534 (8.8)	2507 (49.8)	7957 (36.6)	175 (10.4)	203 (7.7)	722 (16.8)	20601 (31.7)
Other	2581 (22.0)	770 (19.1)	2412 (26.1)	1364 (23.5)	1402 (22.6)	5776 (27.4)	383 (22.2)	721 (27.1)	977 (23.6)	16386 (24.6)
Total	11641 (100.0)	4027 (100.0)	8812 (100.0)	5723 (100.0)	5712 (100.0)	20691 (100.0)	1628 (100.0)	2695 (100.0)	4143 (100.0)	65072 (100.0)
**Community-level exposures**										
**Young women should wait for sex until marriage (reverse coded):**										
Female peer attitudes (aged 15-24 years), N (%):	649 (11.0)	1281 (44.3)	360 (6.2)	845 (21.0)	50 (1.3)	1097 (9.3)	146 (15.9)	96 (3.7)	331 (9.1)	4855 (12.0)
Mean percentage of female peers’ group-attitudes (SD)	10.7 (15.3)	45.8 (18.7)	7.2 (11.7)	22.6 (21.6)	1.5 (7.1)	9.0 (11.6)	17.0 (14.3)	4.2 (8.0)	11.3 (15.1)	12.2 (17.3)
N clusters	744	225	407	497	341	885	104	272	318	3793
Male peer attitudes (aged 15-24 years), N (%):	469 (30.5)	607 (55.4)	160 (12.3)	412 (25.4)	37 (2.8)	689 (13.5)	319 (45.0)	211 (9.8)	337 (12.3)	3241 (19.0)
Mean percentage of male peers’ group-attitudes (SD)	28.3 (36.3)	56.1 (29.5)	12.2 (22.7)	26.7 (31.6)	2.8 (8.9)	14.3 (22.5)	42.0 (25.0)	11.0 (15.0)	14.5 (17.2)	19.6 (27.2)
N clusters	583	216	348	445	292	859	101	271	314	3429
Female adult attitudes (aged 25-49 years), N (%):	839 (7.5)	1406 (36.2)	490 (5.4)	1061 (18.9)	75 (1.2)	1688 (8.4)	239 (16.2)	86 (3.1)	402 (8.1)	6286 (9.7)
Mean percentage of female adult group-attitudes (SD)	7.8 (10.6)	36.9 (19.0)	6.0 (9.9)	20.1 (17.0)	1.4 (4.4)	8.7 (10.1)	15.7 (12.4)	3.2 (6.2)	9.7 (12.4)	10.4 (14.2)
N clusters	750	225	407	500	342	886	104	274	319	3807
Male adult attitudes (aged 25-49 years), N (%):	683 (25.7)	797 (52.6)	152 (6.9)	496 (22.3)	56 (2.2)	1210 (13.3)	441 (40.7)	211 (10.2)	319 (8.5)	4365 (16.7)
Mean percentage of male adult group-attitudes (SD)	24.9 (32.3)	50.8 (25.8)	8.2 (16.7)	23.2 (26.7)	3.1 (7.8)	14.4 (19.5)	39.4 (23.2)	11.3 (15.5)	8.8 (10.5)	17.5 (23.8)
N clusters	727	225	399	483	337	884	103	274	319	3751
**Wife-beating justified if refuse to have sex with her husband:**										
Adult female attitudes (aged 15-49 years), N (%):	3238 (17.7)	2434 (36.9)	7862 (59.3)	1254 (12.6)	5172 (58.8)	8952 (26.6)	150 (5.9)	172 (3.4)	2556 (38.5)	31790 (30.9)
Mean of group-attitudes (SD)	18.9 (18.1)	36.2 (16.2)	56.3 (23.9)	13.5 (14.6)	58.1 (26.2)	28.2 (22.3)	6.1 (7.1)	3.6 (4.9)	38.2 (19.4)	31.2 (26.0)
N clusters	1331	448	803	835	672	1685	169	388	633	6964

SD – standard deviation

### Variables

#### Outcome: contraceptive use and demand satisfied

The main outcomes for this study were the use of, and demand satisfied for, contraception. Assessment of contraceptive use was conducted using individual responses from a sample of 73 090 sexually-active fecund women of reproductive age (15-49 years) that included women with no unmet need – defined as not using contraception and not desiring to space or limit new pregnancies – and with demand for family planning, defined as the sum of contraceptive use and unmet need for family planning. Assessment of demand satisfied for contraception was conducted using a restricted sample of 41 625 women with demand for family planning (see **Figure 1**). Our estimates for contraceptive use/demand satisfied relied on DHS information regarding the current use of contraceptive methods. For women not using contraception, we applied a standard definition of unmet need, a simplified way to obtain consistent estimates over time and across countries/surveys [41]. This standard definition was based on survey information of current fertility and fertility preferences, including pregnancy or postpartum amenorrhoeic status, desire to limit childbearing, ideal number of children, and spacing (see Appendix S1 and Figure S1 in the **Online Supplementary Document** for a glossary of terms used in its definition and a detailed description of unmet need estimation, respectively). This definition also allowed us to include sexually active unmarried women, who have been traditionally excluded from estimates and analyses of unmet need [41].

#### Exposure: community-level proxies for gender norms

The DHS surveys included questions about attitudes towards sex before marriage asked of male and female respondents (aged 15-49 years) in the nine countries in our sample. We reverse-coded data on whether male or female respondents think ‘young women should wait for sex until marriage’ to operationalise permissive attitudes at the individual level, classifying respondents as permissive if they believed that there is ‘no need for young women to wait for sex until marriage’. At the community level, collective permissive attitudes among male or female peers (aged 15-24 years) or adults (aged 25-49 years) were approximated as the percentage of peers or adults living in the same community who believed that young women do not need to wait for sex until marriage. Similarly, individual attitudes towards violence were based on respondents’ justification of violence when a wife refuses to have sex with her husband; the question was asked of women and men, but we only used women’s responses [30]. Collective accepting attitudes towards wife-beating were approximated at the community level as the percentage of females aged 15-49 years living in the same community who admitted that wife beating is justifiable when she refuses to have sex with her husband. An additional four items available in DHS that justify wife-beating (going out without telling her husband; arguing with her husband; neglecting the children; and burning the food) were also tested but excluded from the final analysis because they were highly correlated and produced contradicting associations. We included both individual- and community-level attitudes in our models to control for compositional effects and the proper assessment of community-level effects of attitudinal norms towards premarital sex and wife-beating. In this analysis, communities were defined as the area-based PSUs or clusters as reported in the DHS survey for each country in our sample [40]. The design of PSUs facilitate comparative analysis within and across countries because they were roughly equal in population and in many instances they corresponded to villages or clusters of nearby villages [42,43].

#### Covariates

**Figure 2:**
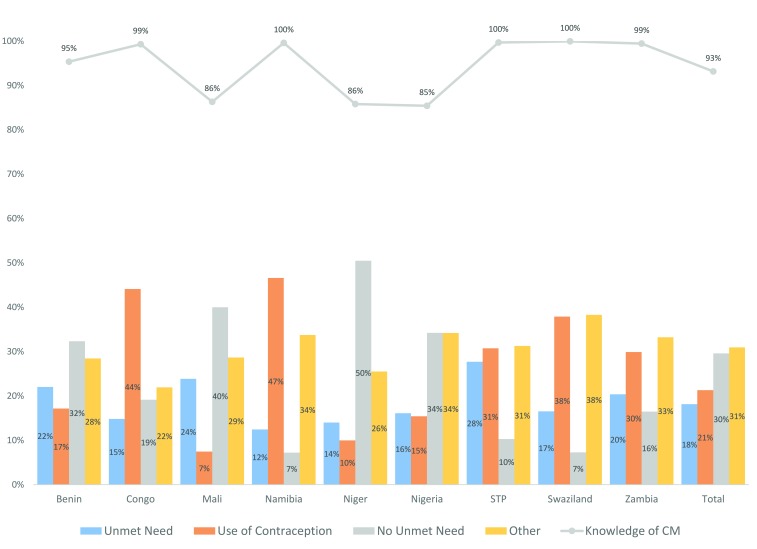
Percentage of women aged 15-49 years with unmet need, contraceptive prevalence, no unmet need, and other; and with knowledge of contraceptive methods in nine SSA countries (2005-2009). STP – Sao Tome and Principe, CM – contraceptive method. ‘Other’ includes infecund or menopausal, not sexually active women in reproductive age, and missing values. The correlation between unmet need and knowledge of contraception methods was -0.29 (Spearman Correlation (Sr) = -0.29, *P* < 0.001).

In our statistical analysis, we controlled for individual background characteristics and sociodemographic status of our study population, including age, marital status, level of education, work status, parity, type of residency, and wealth status. Age was recorded in single years (ages 15-49); marital status was categorised in three groups: formerly married, never married, and currently married; education was categorised as primary, secondary, or higher; work was coded as 1 if the respondent was currently working and 0 otherwise; parity was defined as the number of live children ever born to each women and categorised in 4 groups (1, 2, 3, 4 or more); and residency was split in rural and urban. Wealth was specified in quintiles, available in DHS surveys at the household level and constructed from a wealth index that reflects differences in the ownership of household assets [44]. In models with pooled data, we additionally controlled for country fixed-effects. Knowledge of contraceptive methods was included in the descriptive analysis, but not as a covariate for the inference analysis because of the very high prevalence observed in most countries (**Figure 2**).

In augmented models, we further controlled for three community-level factors that reflect gender empowerment as shown in previous studies [30]: women’s educational achievement, early marriage, and job status. Educational achievement was defined as the proportion of women aged 15-49 years who completed secondary or higher education; early marriage represents the proportion of women aged 20-24 years who were married before age 18; and employment was defined as the percentage of women aged 15-49 years in the community who were currently employed at the time of the survey [30].

### Statistical analysis

#### Descriptive analysis

Simple and cross tabulations and weighted proportions were estimated to assess the distribution of women’s characteristics according to their age, fertility and family planning preferences. We estimated the prevalence of contraception use and demand satisfied by country.

#### Inferential analysis

We fitted age-stratified logistic two-level multilevel (ML) random intercept models for binary outcomes, controlling for individual-level sociodemographic factors and community-level factors reflecting women’s empowerment, to test the association of individual use of, and demand satisfied for, contraception with permissive attitudes towards premarital sex and accepting attitudes of wife-beating at the community level. We only considered attitudes of women as exposure in the case of wife-beating, but for premarital sex we additionally considered group-level attitudes of both women or men (in the groupings of peers and adults) as exposures at the community level. We conducted separate analyses for each outcome, denoting as Model A the analysis for use of contraception, and Model B for demand satisfied with any contraceptive method, as illustrated in **Figure 1**.

##### Model A: Use of contraception

We split the pooled sample of 73 090 sexually-active fecund women into two groups of 24 404 adolescents (aged 15-24 years) and 48 686 adults (aged 25-49 years) to conduct separate multilevel analyses of contraceptive use. For the sample of adolescents, we ran a set of 9 separate model specifications, starting with an unadjusted ML model that controlled only for random effects and country fixed-effects (Model 0 [M0]). We then tested the separate effects of collective attitudes towards premarital sex (Model 1 [M1]) and wife-beating (Model 3 [M3]), and also controlling for the corresponding individual attitudes to account for compositional effects (Model 2 [M2] & Model 4 [M4], respectively). The joint effect of attitudinal norms towards premarital sex and wife-beating was tested in Model 5 [M5]. We further controlled for collective and individual predictors of women’s empowerment: women’s educational attainment, early marriage, and work status (Model 6 [M6]; and ended with fully adjusted models that additionally included individual-level covariates: age, parity, wealth, residency (fully adjusted models 1 [FA1] and 2 [FA2]). This procedure was conducted to assess the separate effects and the community-level variation attributed to each subset of individual and community-level predictors and covariates. A similar approach was followed with the subsample of adult women, as we illustrate in **Figure 1****,** which also reports the sample sizes in the age-stratified pooled models.

##### Model B: Demand satisfied with any contraceptive method

The analysis of demand satisfied was conducted using the reduced sample of 41 625 women with any demand for contraception. We also split the sample into two groups of 13 540 adolescents (aged 15-24 years) and 28 085 adults (aged 25-49) to conduct separate multilevel analyses in each group. We tested the same set of model specifications as for Model A (M0-M6, FA1, and FA2), as shown in **Figure 1**.

##### Country-specific analysis and effect modification

All sets of (type A and B) models were fitted separately for each country, and we performed the Mantel-Haenszel (MH) homogeneity test to assess whether the exposure effect was the same across countries in our pooled model specification [45]. The MH statistic was estimated separately for each exposure variable of attitudes towards premarital sex and wife-beating.

##### Assessment of community-level effects

Models A and B examined the specific community-level (effect-size) associations between contraceptive use and demand satisfied, and collective attitudinal norms, in pooled samples and across countries. To further determine the relative significance of those associations, we assessed the general community-level effects that we measured using the intra-class correlation (ICC) [46], defined as the proportion of the residual variance of the outcome (use or demand satisfied for contraception) attributed to the community level (V_2_), ie, the ratio of between-community variance to the total. Furthermore, we measured the percentage of community-level attributed variance explained (VE) by subsets of individual or community-level exposures [42,43], as defined above in our 9 separate model specifications (M0-M6, FA1, or FA2), relative to unadjusted models with no covariates or risk factors (M0) (see Appendix S2 in the **Online Supplementary Document** for details about this methodology).

Multilevel models are suitable for the efficient estimation of random effects at different levels of aggregation [47], but in models with binary outcomes, random effects at the individual level are not measured directly but we estimated them using a latent variable formulation [48] (Appendix S2 in the **Online Supplementary Document**). To facilitate the interpretation of collective (group-mean) variables, all community-level exposures and covariates were standardised to have mean 0 and standard deviation (SD) 1 within each country. For the estimation of means and/or proportions, we accounted for the complex study design of DHS surveys, which includes unequal probabilities of selection, cluster sampling, and stratification of the target population [49]. All analyses were conducted using Stata/SE software version 14.2 (StataCorp LLC, College Station TX, USA) [50].

### Ethical review

DHS data are publicly available and contain no personal identifiable information. Ethical clearances were obtained by the ORC Macro Institutional Review Board, as well as by individual review boards within each participating country, when data were originally collected. Stanford IRB approval was obtained through the Stanford Population Health Sciences Center IRB #42971: Lancet Series on Gender and Health.

## RESULTS

### Descriptive analysis

The total demand for family planning among all women aged 15-49 years varied significantly across countries, from 24% in Niger to 59% in Congo, Namibia, and Sao Tome and Principe (STP). Congo and Namibia were also the countries with the highest contraceptive prevalence (44% and 47%, respectively), while Mali and STP were the countries with the highest percentage of women with unmet need (24% and 28%, respectively). Four countries showed high percentages (>30%) of women with no unmet need, ranging from 32% in Benin to 50% in Niger (**Figure 2**). We found an inverse association between unmet need and the degree of contraception knowledge (Spearman Correlation (Sr) = -0.29, *P* < 0.001), a potential predictor of high unmet need, in some countries; Mali, Niger and Nigeria reported the highest prevalence of unmet need and the lowest prevalence of contraception knowledge (**Figure 2**).

In the pooled sample, younger women’s attitudes towards premarital sex were slightly more permissive than those of older women. The percentage of young women aged 15-24 years with permissive attitudes for premarital sex was 12.0%, in comparison with 9.7% among adult women aged 25-49 years. Compared to women’s, men’s attitudes were more permissive, although with a similar gap between both age groups (with respective percentages of 19.0% and 16.7% for young and adult men). A similar pattern was observed across countries, but with marked heterogeneities; the percentage of young women with permissive attitudes varied from 1.3% in Niger to 44.3% in Congo. In general, we observed a right-skewed distribution of permissive attitudes (eg, as reflected by mean percentage of female peers’ group-attitudes = 12.2% and SD = 17.3, **Table 1**), with proportions around or below 10% in most countries (Benin, Mali, Niger, Nigeria, Swaziland, and Zambia); this is reflected in the distribution of this exposure at the community level (**Table 1**). Among women aged 15-49 years, the proportion of women with accepting attitudes towards wife-beating was 30.9% in the pooled sample, and it also varied substantially across countries, from 3.4% in Swaziland to 58.8% in Niger (**Table 1**).

### Model A: Contraceptive use

In pooled samples of fecund 24 404 adolescent women (aged 15-24 years), the odds of contraceptive use increased by 22% with a 1 SD increase in the variation of collective attitudes towards premarital sex among female peers (odds ratio (OR) = 1.22, 95% CI = 1.15-1.30) and increased 12% with a 1 SD increase in the variation of collective attitudes towards premarital sex among male peers (OR = 1.12, 1.06-1.19) (M1, **Table 2**). The effect of female peer’s attitudes declined to 16% (OR = 1.16, 95% CI = 1.09-1.24) after controlling for individual permissive attitudes towards premarital sex of adolescent women, but the effect of male peer’s attitudes remained stable (OR = 1.13, 95% CI = 1.07-1.20) (M2). In a separate model (M3), collective accepting attitudes towards wife-beating were associated with a lower odds of contraception use (OR = 0.56, 95% CI = 0.53-0.60), but the effect was slightly attenuated after further controlling for individual attitudes towards wife-beating (OR = 0.59, 95% CI = 0.55-0.63) (M4). The combined effect of attitudinal norms towards premarital sex and wife-beating only reduced the effect of adolescent female peers’ attitudes (OR = 1.12, 95% CI = 1.05-1.19) on the contraception use of adolescent women (M5, **Table 2**).

**Table 2:** Model A: Association of collective (peer and adult) permissive attitudes towards premarital sex and acceptance of wife-beating, and the individual use of contraception using logistic two-level multilevel random intercept models for adolescent (aged 15-24 years) and adult (aged 15-24 years) women in a pooled sample of nine SSA countries.

Outcome:	Adolescent women (aged 15-24 years)								Adult women (aged 25-49 years)
**Use of contraception**	**M1:**	**M2:**	**M3:**	**M4:**	**M5:**	**M6:**	**FA1:**	**FA2:**	**FA2:**
	**collective attitudes towards premarital sex**	**M1 + individual attitudes towards premarital sex**	**collective attitudes towards wife-beating**	**M3 + individual attitudes towards wife-beating**	**M2 + M4**	**M5 + women's empowerment**	**M6 + individual level covariates (peers' attitudes in M1)**	**M6 + individual level covariates (adults' attitudes in M1)**	**M6 + individual level covariates (adults' attitudes in M1)**
**Community-level variables (OR per 1 SD increase, 95% CI)**									
**Collective attitudinal norms:**									
Acceptance of premarital sex (1 SD):									
Female peer	1.22 (1.15-1.30)	1.16 (1.09-1.24)			1.12 (1.05-1.19)	1.08 (1.02-1.14)	1.08 (1.02-1.15)		
Male peer	1.12 (1.06-1.19)	1.13 (1.07-1.20)			1.12 (1.06-1.19)	1.10 (1.04-1.16)	1.11 (1.05-1.17)		
Female adult								1.07 (1.01-1.12)	1.04 (1.00-1.08)
Male adult								1.06 (1.01-1.11)	1.08 (1.04-1.12)
Acceptance of wife-beating (1 SD)			0.56 (0.53-0.60)	0.59 (0.55-0.63)	0.60 (0.56-0.64)	0.89 (0.84-0.95)	0.90 (0.85-0.96)	0.91 (0.86-0.97)	0.88 (0.84-0.93)
Women's empowerment:*									
Secondary/higher school completion (1 SD)						1.46 (1.37-1.57)	1.31 (1.21-1.42)	1.30 (1.21-1.41)	1.36 (1.29-1.44)
Early marriage (1 SD)						0.96 (0.91-1.01)	0.96 (0.91-1.02)	0.97 (0.91-1.03)	0.91 (0.88-0.95)
Currently working						1.03 (0.97-1.10)	1.03 (0.97-1.10)	1.04 (0.98-1.10)	1.11 (1.06-1.17)
**Individual-level variables (OR, 95% CI):**									
Individual permissive attitudes towards premarital sex		1.38 (1.21-1.58)			1.38 (1.21-1.58)	1.21 (1.04-1.39)	1.24 (1.07-1.43)	1.25 (1.09-1.44)	1.00 (0.89-1.11)
Individual accepting attitudes towards wife-beating				0.79 (0.70-0.88)	0.77 (0.69-0.87)	0.85 (0.75-0.96)	0.85 (0.75-0.96)	0.87 (0.78-0.99)	1.00 (0.92-1.08)
Level of education (None = 1):									
Primary						2.08 (1.79-2.43)	2.17 (1.86-2.53)	2.20 (1.89-2.55)	1.75 (1.59-1.91)
Secondary						3.07 (2.61-3.63)	3.20 (2.70-3.79)	3.29 (2.79-3.87)	2.26 (2.02-2.53)
Higher						5.89 (4.29-8.11)	5.18 (3.70-7.26)	5.43 (3.90-7.55)	2.98 (2.51-3.55)
Marital status:									
Never in union						4.32 (3.81-4.90)	5.76 (4.98-6.66)	5.88 (5.10-6.79)	6.43 (5.24-7.89)
Formerly in union						2.10 (1.56-2.82)	2.14 (1.59-2.90)	2.14 (1.59-2.86)	2.36 (2.02-2.74)
Currently working						1.25 (1.12-1.40)	1.16 (1.04-1.30)	1.14 (1.03-1.27)	1.20 (1.11-1.30)
Age							1.07 (1.05-1.10)	1.07 (1.05-1.10)	1.01 (1.01-1.02)
Number of live births (No children = 1):									
1							1.05 (0.92-1.21)	1.05 (0.92-1.20)	1.41 (1.16-1.71)
2							1.52 (1.29-1.78)	1.52 (1.30-1.78)	1.98 (1.63-2.40)
3							1.64 (1.31-2.05)	1.64 (1.32-2.04)	2.57 (2.12-3.12)
4 or more							1.41 (1.05-1.89)	1.38 (1.04-1.84)	3.13 (2.58-3.79)
Wealth quintile (Q1 = 1):									
Q2							1.00 (0.84-1.18)	1.01 (0.86-1.19)	1.11 (1.00-1.24)
Q3							1.00 (0.83-1.19)	1.04 (0.87-1.23)	1.29 (1.15-1.45)
Q4							1.23 (1.02-1.49)	1.28 (1.06-1.53)	1.76 (1.56-1.98)
Q5							1.47 (1.18-1.83)	1.55 (1.25-1.91)	2.14 (1.86-2.47)
Residency (Rural = 1):									
Urban							1.05 (0.91-1.20)	1.02 (0.89-1.17)	0.97 (0.88-1.07)
Observations	22,510	21,491	24,404	23,592	20,832	19,441	19,441	20,942	40,834
Number of groups	3,382	3,376	3,744	3,736	3,371	2,950	2,950	3,235	3,248

M1-M6 – models 1 to 6, FA1 – fully-adjusted Model 1, FA2 – fully-adjusted model 2, OR – odds ratio, SD – standard deviation, CI – confidence interval.

*For adolescent women, models are presented adjusting for peers’ collective attitudes (FA1 model) and adults’ collective attitudes (FA2 model). We additionally controlled for country fixed-effects in all models.

†Educational achievement was defined as the proportion of women aged 15-49 years who completed secondary or higher education; early marriage represents the proportion of women aged 20-24 years who were married before age 18; and employment was defined as the percentage of women aged 15-49 years in the community who were currently employed at the time of the survey.

The effects of collective adolescent peers’ attitudes (female: OR = 1.08, 95% CI = 1.02-1.14; male: OR = 1.10, 95% CI = 1.04-1.16) and acceptance of wife-beating (OR = 0.89, 95% CI = 0.84-0.95) were attenuated after further controlling for collective and individual indicators of women’s empowerment. Among these indicators, the odds of contraception only increased with a higher variation of collective women’s educational achievement (OR = 1.46, 95% CI = 1.37-1.57). At the individual level, the odds of contraception increased with educational level [eg, women with secondary education relative to those who were uneducated (OR = 3.07, 95% CI = 2.61-3.63)], for women never in union (OR = 4.32, 95% CI = 3.81-4.90) relative to married women, and for currently working women (OR = 1.25, 1.12-1.40) (M6).

Finally, in fully-adjusted models that further controlled for individual level covariates (FA1), the effect of collective attitudes of adolescent peers (female: OR = 1.08, 95% CI = 1.02-1.15; male: 1.11, 95% CI = 1.05-1.17) and acceptance of wife-beating (OR = 0.90, 95% CI = 0.85-0.96) remained unchanged. The odds of contraceptive use increased with age (OR = 1.07, 1.05-1.10), after the first birth (eg, women with 3 children ever born alive (OR = 1.64, 95% CI = 1.31-2.05) in comparison with women with no births), and with wealth status, eg, women in quintile 5 relative to women in quintile 1 (OR = 1.47, 95% CI = 1.18-1.83) (FA1, **Table 2**). In a separate fully adjusted model (FA2), contraceptive use was also associated with a higher variation of collective attitudinal norms towards premarital sex of adult women (OR = 1.07, 95% CI-1.01-1.12) and men (OR = 1.06, 95% CI = 1.01-1.11) aged 25-49 years.

In another separate fully-adjusted model (FA2) for adult women (aged 25-49 years) outcomes (N = 40 834), we found statistically significant effects in the variation of collective attitudinal norms of female (OR = 1.04, 95% CI = 1.00-1.08) and male (OR = 1.08, 95% CI 1.04-1.12) adults regarding premarital sex, and women’s acceptance of wife-beating (OR = 0.88, 95% CI = 0.84-0.93), as well as for women’s collective educational achievement (OR = 1.36, 95% CI = 1.29-1.44), early marriage (OR = 0.91, 95% CI = 0.88-0.95), and currently working women (OR = 1.11, 95% CI = 1.06-1.17). The odds of contraceptive use was also higher for most individual level predictors (education, unmarried women, work status, parity, and wealth status), but not for individual level attitudes (**Table 2**).

### Model B: Total demand satisfied with any contraceptive method

In fully-adjusted models in a subsample of 13 540 fecund adolescent women (aged 15-24) with any demand for family planning, a 1 SD increase of the variance in the proportion of adolescent women peers (aged 15-24 years) or adults (aged 25-49 years) with collective permissive attitudes toward premarital sex increased the odds of satisfied demand of adolescent women with any contraceptive method by 6% (peers: OR = 1.06, 95% CI = 0.99-1.13) and by 7% (adults: OR = 1.07, 95% CI = 1.01-1.13), respectively. Collective accepting attitudes towards wife-beating among women (aged 15-49 years) had negative associations with the demand satisfied with any method among adolescent women (OR = 0.89, 95% CI = 0.83-0.95) (**Table 3**). In a subsample of adult women (aged 25-49, N=22 765), the odds of demand satisfied with any method also decreased significantly with increments in the variation of collective attitudes towards wife-beating (OR = 0.86, 95% CI = 0.81-0.90) and with increases in the variability of women achieving higher levels of education (OR = 1.24, 95% CI = 1.16-1.32), early marriage (OR = 0.95, 95% CI = 0.91-0.99), or being employed (OR = 1.10, 95% CI = 1.05-1.16) (**Table 3** and Table S1 in the **Online Supplementary Document**).

**Table 3:** Model B: Association of collective (peer and adult) permissive attitudes towards premarital sex and acceptance of wife-beating, and the individual demand satisfied with any contraceptive method using logistic two-level multilevel random intercept models for adolescent (aged 15-24 years) and adult (25-49 years) women in pooled samples of nine SSA countries*

Outcome: Demand satisfied	Adolescent women (aged 15-24 years), OR (95% CI)		Adult women (aged 25-49 years), OR (95% CI)
	**FA1**	**FA2**	**FA2**
**Community-level variables (OR per 1 SD increase, 95% CI)**			
**Collective attitudinal norms**			
Acceptance of premarital sex (1 SD):			
Female peer	1.06, (0.99-1.13)		
Male peer	1.07 (1.01-1.13)		
Female adult		1.06 (1.00-1.12)	1.04 (0.99-1.08)
Male adult		1.02 (0.97-1.08)	1.04 (0.99-1.09)
Acceptance of wife-beating (1 SD):			
	0.89	0.89	0.86
	(0.83-0.95)	(0.83-0.96)	(0.81-0.90)
Women's empowerment:†			
Secondary/higher school completion (1 SD)	1.16	1.17	1.24
	(1.06-1.27)	(1.07-1.27)	(1.16-1.32)
Early marriage (1 SD)	1.01	1.02	0.95
	(0.95-1.08)	(0.95-1.08)	(0.91-0.99)
Currently working	1.02	1.02	1.1
	(0.95-1.09)	(0.95-1.09)	(1.05-1.16)
Observations	10,408	11,072	22765
Number of groups	2,678	2,914	3201

FA1 – fully-adjusted Model 1, FA2 – fully-adjusted Model 2, SD – standard deviation, OR – odds ratio, CI – confidence interval.

* The model specifications of FA1 and FA2 are described in **Figure 1** and used in **Table 2**. We controlled for individual attitudes and sociodemographic characteristics: age, parity, marital status, education, work status, wealth status, place of residence, and country fixed-effects. For adolescent women, models are presented adjusting for peers’ collective attitudes (FA1 model) and adults’ collective attitudes (FA2 model).

†Educational achievement was defined as the proportion of women aged 15-49 years who completed secondary or higher education; early marriage represents the proportion of women aged 20-24 years who were married before age 18; and employment was defined as the percentage of women aged 15-49 years in the community who were currently employed at the time of the survey.

### Country-specific results and effect modification

Replicating the previous analyses on a country-basis revealed heterogenous effects across countries in the association of contraceptive use and community-level effects of attitudes towards premarital sex among adolescent women (Mantel-Haenszel χ^2^ (MH) = 85.68, *P* < 0.001) and adult women (MH = 8.83, *P* < 0.01), as well as for wife-beating accepting attitudes among adolescent (MH = 260.25, *P* < 0.001) and adult (MH = 309.68, *P* < 0.001) women (**Table 4**). In fully adjusted models (FA1), we found positive associations of collective permissive attitudes of adolescent women peers with contraceptive use in four countries, but they were significant only in Namibia (OR = 1.29, 95% CI = 1.06-1.57) and Nigeria (OR = 1.13, 95% CI = 1.01-1.27). Significant negative associations of collective accepting attitudes towards wife-beating with contraceptive use were found in Benin (OR = 0.74, 95% CI = 0.61-0.89), Nigeria (OR = 0.80, 95% CI = 0.66-0.96), and Zambia (OR = 0.77, 95% CI = 0.65-0.90). Furthermore, significant associations were also observed in Nigeria in models examining collective women’s educational achievement (OR = 1.77, 95% CI = 1.40-2.24) and early marriage (OR = 0.86, 95% CI = 0.75-0.99), and significant associations were found for collective women’s working status only in Namibia (OR = 1.27, 95% CI = 1.05-1.54) (**Table 4**).

**Table 4:** Models A & B: Association of collective (peer and adult) permissive attitudes towards premarital sex and adult acceptance of wife-beating, and the individual use of (model A), and demand satisfied for (model B), contraception using logistic two-level multilevel random intercept models for adolescent (aged 15-24 years) and adult (25-49 years) women. Pooled and country-specific analysis of nine SSA countries*

		Gender norms					Women's empowerment† (FA1)		
	**Acceptance of premarital sex**				**Acceptance of wife-beating**	**Secondary/higher school completion**	**Early marriage**	**Currently working**
**N**	**Peers (FA1)**		**Adults (FA2)**					
	**Female**	**Male**	**Female**	**Male**				
**Use of contraception (ORs per 1 SD increase, 95% CI)**									
**Youth women aged 15-24 years:**									
Pooled sample	19 441	1.08 (1.02-1.15)	1.11 (1.05-1.17)	1.07 (1.01-1.12)	1.06 (1.01-1.11)	0.90 (0.85-0.96)	1.31 (1.21-1.42)	0.96 (0.91-1.02)	1.03 (0.97-1.10)
Benin	2682	0.90 (0.76-1.06)	1.41 (1.23-1.61)	0.98 (0.88-1.10)	1.33 (1.17-1.50)	0.74 (0.61-0.89)	1.15 (0.92-1.43)	0.94 (0.81-1.10)	1.06 (0.91-1.24)
Congo	1920	1.10 (0.94-1.29)	1.00 (0.87-1.14)	1.10 (0.97-1.25)	1.06 (0.93-1.20)	1.08 (0.93-1.25)	1.14 (0.92-1.41)	1.14 (1.01-1.30)	0.92 (0.76-1.11)
Mali	3120	1.00 (0.80-1.25)	1.06 (0.87-1.29)	1.05 (0.88-1.25)	1.06 (0.91-1.23)	1.17 (0.92-1.49)	1.22 (0.97-1.54)	1.04 (0.86-1.25)	0.79 (0.63-0.99)
Namibia	1143	1.29 (1.06-1.57)	1.04 (0.90-1.21)	1.14 (0.98-1.32)	0.98 (0.86-1.13)	0.94 (0.80-1.11)	1.09 (0.88-1.35)	1.07 (0.90-1.27)	1.27 (1.05-1.54)
Niger	1726	0.88 (0.56-1.39)	1.03 (0.80-1.33)	0.82 (0.61-1.12)	0.95 (0.74-1.21)	1.64 (1.13-2.37)	1.03 (0.71-1.51)	0.86 (0.61-1.21)	1.18 (0.88-1.58)
Nigeria	6054	1.13 (1.01-1.27)	1.03 (0.92-1.16)	1.05 (0.93-1.19)	1.02 (0.90-1.15)	0.80 (0.66-0.96)	1.77 (1.40-2.24)	0.86 (0.75-0.99)	1.13 (0.96-1.32)
STP	450	0.86 (0.61-1.22)	0.97 (0.74-1.27)	0.95 (0.74-1.21)	0.82 (0.64-1.06)	0.90 (0.63-1.29)	1.06 (0.72-1.56)	1.04 (0.78-1.39)	1.17 (0.87-1.58)
Swaziland	850	0.97 (0.81-1.15)	0.94 (0.78-1.13)	1.06 (0.89-1.25)	0.81 (0.68-0.96)	0.92 (0.77-1.10)	0.94 (0.74-1.18)	1.07 (0.89-1.29)	0.92 (0.74-1.13)
Zambia	1496	1.05 (0.89-1.23)	1.08 (0.89-1.32)	1.06 (0.90-1.24)	0.94 (0.83-1.06)	0.77 (0.65-0.90)	1.17 (0.93-1.48)	1.08 (0.93-1.26)	0.87 (0.75-1.00)
Mantel-Haenszel χ^2^		85.68 (*P* < 0.001)				260.25 (*P* < 0.001)			
**Adult women aged 25-49 years:**									
Pooled sample	40 834			1.04 (1.00-1.08)	1.08 (1.04-1.12)	0.88 (0.84-0.93)	1.36 (1.29-1.44)	0.91 (0.88-0.95)	1.11 (1.06-1.17)
Benin	7712			0.99 (0.90-1.09)	1.26 (1.15-1.37)	0.77 (0.68-0.87)	1.28 (1.11-1.48)	1.01 (0.92-1.11)	1.20 (1.06-1.36)
Congo	2916			1.12 (0.99-1.26)	1.06 (0.96-1.16)	1.13 (1.01-1.27)	1.02 (0.85-1.21)	1.01 (0.91-1.12)	0.97 (0.82-1.14)
Mali	5790			1.05 (0.92-1.21)	1.03 (0.93-1.15)	1.05 (0.92-1.21)	1.40 (1.19-1.65)	1.05 (0.93-1.19)	1.08 (0.91-1.28)
Namibia	2093			1.04 (0.92-1.17)	0.95 (0.85-1.05)	0.97 (0.86-1.09)	1.22 (1.04-1.42)	1.01 (0.91-1.12)	1.12 (0.98-1.28)
Niger	4003			0.90 (0.76-1.08)	0.99 (0.85-1.15)	1.86 (1.51-2.30)	1.38 (1.11-1.71)	0.81 (0.70-0.93)	0.89 (0.74-1.07)
Nigeria	13 103			1.06 (0.97-1.15)	1.04 (0.96-1.13)	0.71 (0.62-0.80)	1.72 (1.49-1.99)	0.84 (0.77-0.92)	1.24 (1.13-1.37)
STP	1013			1.10 (0.93-1.29)	0.96 (0.79-1.17)	0.96 (0.78-1.19)	0.87 (0.71-1.06)	1.02 (0.84-1.24)	1.07 (0.89-1.28)
Swaziland	1280			1.19 (1.05-1.36)	0.96 (0.81-1.13)	0.97 (0.85-1.10)	1.11 (0.92-1.35)	1.01 (0.89-1.15)	1.03 (0.87-1.22)
Zambia	2916			0.95 (0.84-1.08)	0.87 (0.78-0.97)	0.70 (0.61-0.79)	1.10 (0.91-1.33)	1.02 (0.91-1.15)	0.93 (0.81-1.06)
Mantel-Haenszel χ^2^		8.83 (*P* < 0.01)				309.68 (*P* < 0.001)			
**Demand satisfied (ORs per 1 SD increase, 95% CI)**									
**Youth women aged 15-24 years:**									
Pooled sample	10 408	1.06 (0.99-1.13)	1.07 (1.01-1.13)	1.06 (1.00-1.12)	1.02 (0.97-1.08)	0.89 (0.83-0.95)	1.16 (1.06-1.27)	1.01 (0.95-1.08)	1.02 (0.95-1.09)
Benin	1414	0.90 (0.74-1.09)	1.36 (1.17-1.60)	1.01 (0.88-1.16)	1.26 (1.08-1.46)	0.73 (0.58-0.92)	1.07 (0.81-1.40)	1.01 (0.84-1.22)	1.10 (0.90-1.33)
Congo	1607	1.11 (0.91-1.34)	0.95 (0.81-1.13)	1.03 (0.88-1.22)	1.00 (0.85-1.17)	1.03 (0.85-1.26)	1.11 (0.84-1.46)	1.15 (0.98-1.35)	0.94 (0.72-1.21)
Mali	1293	0.89 (0.70-1.14)	1.02 (0.83-1.27)	0.98 (0.81-1.19)	1.01 (0.85-1.19)	1.12 (0.87-1.43)	1.07 (0.82-1.38)	1.13 (0.92-1.38)	0.84 (0.64-1.10)
Namibia	1003	1.18 (0.92-1.51)	1.21 (1.01-1.44)	1.17 (0.98-1.39)	1.08 (0.91-1.27)	1.00 (0.83-1.20)	1.15 (0.89-1.48)	1.11 (0.91-1.35)	1.32 (1.05-1.67)
Niger	551	0.81 (0.49-1.32)	0.92 (0.66-1.27)	0.89 (0.67-1.16)	1.16 (0.86-1.57)	1.34 (0.90-2.00)	0.93 (0.57-1.53)	1.03 (0.71-1.49)	1.20 (0.85-1.69)
Nigeria	2421	1.14 (1.00-1.29)	1.00 (0.88-1.14)	1.06 (0.93-1.22)	0.97 (0.84-1.11)	0.83 (0.67-1.03)	1.75 (1.32-2.30)	0.86 (0.74-1.01)	1.08 (0.90-1.30)
Sao Tome and Principe	355	0.81 (0.54-1.20)	1.07 (0.78-1.45)	1.03 (0.80-1.31)	0.84 (0.62-1.14)	1.00 (0.67-1.50)	1.15 (0.75-1.74)	0.98 (0.71-1.35)	1.22 (0.90-1.65)
Swaziland	719	0.98 (0.79-1.20)	0.91 (0.76-1.10)	1.06 (0.88-1.27)	0.79 (0.66-0.95)	0.90 (0.76-1.07)	0.88 (0.69-1.13)	1.02 (0.84-1.23)	0.96 (0.76-1.21)
Zambia	1045	1.19 (0.98-1.44)	1.04 (0.83-1.31)	1.13 (0.93-1.37)	1.02 (0.86-1.21)	0.75 (0.62-0.91)	1.22 (0.92-1.61)	1.16 (0.96-1.41)	0.86 (0.72-1.04)
Mantel-Haenszel χ^2^		33.20 (*P* < 0.001)				107.03 (*P* < 0.001)			
**Adult women aged 25-49 years:**									
Pooled sample	22 765			1.04 (0.99-1.08)	1.04 (0.99-1.09)	0.86 (0.81-0.90)	1.24 (1.16-1.32)	0.95 (0.91-0.99)	1.1 (1.05-1.16)
Benin	4161			0.95 (0.85-1.07)	1.20 (1.09-1.33)	0.79 (0.69-0.89)	1.19 (1.00-1.40)	1.07 (0.97-1.19)	1.20 (1.04-1.37)
Congo	2125			1.12 (0.97-1.28)	1.01 (0.89-1.15)	1.05 (0.91-1.22)	0.96 (0.75-1.23)	1.11 (0.98-1.27)	1.10 (0.87-1.38)
Mali	2680			0.97 (0.85-1.12)	0.99 (0.86-1.14)	1.04 (0.89-1.21)	1.25 (1.02-1.52)	1.08 (0.95-1.23)	1.02 (0.85-1.22)
Namibia	1816			1.08 (0.94-1.25)	0.90 (0.78-1.03)	1.00 (0.86-1.17)	1.29 (1.06-1.56)	1.04 (0.92-1.19)	1.07 (0.91-1.26)
Niger	1666			1.01 (0.79-1.31)	0.96 (0.81-1.15)	1.67 (1.28-2.17)	1.43 (1.11-1.84)	0.81 (0.68-0.97)	0.87 (0.70-1.06)
Nigeria	6037			1.08 (0.98-1.19)	1.00 (0.90-1.10)	0.69 (0.60-0.80)	1.46 (1.23-1.72)	0.83 (0.75-0.93)	1.21 (1.09-1.35)
STP	868			1.07 (0.89-1.28)	0.96 (0.77-1.20)	0.98 (0.78-1.24)	0.84 (0.66-1.08)	1.05 (0.83-1.33)	1.06 (0.85-1.31)
Swaziland	1143			1.14 (0.97-1.32)	0.96 (0.79-1.18)	0.93 (0.80-1.09)	1.12 (0.90-1.39)	0.99 (0.85-1.16)	0.92 (0.76-1.12)
Zambia	2258			1.04 (0.90-1.19)	0.90 (0.79-1.02)	0.68 (0.59-0.77)	1.06 (0.86-1.30)	0.99 (0.87-1.13)	0.95 (0.82-1.11)
Mantel-Haenszel χ^2^		5.43 (*P* < 0.05)				234.05 (*P* < 0.001)			

FA1 – fully-adjusted Model 1, FA2 – fully-adjusted Model 2, OR – odds ratio, SD – standard deviation, CI – confidence interval.

*The model specifications of FA1 and FA2 are described in **Figure 1** and used in **Table 2**. We controlled for individual attitudes and sociodemographic characteristics: age, parity, marital status, education, work status, wealth status, place of residence, and country fixed-effects (pooled sample). For adolescent women, models are presented adjusting for peers’ collective attitudes (FA1 model) and adults’ collective attitudes (FA2 model).

†Educational achievement was defined as the proportion of women aged 15-49 years who completed secondary or higher education; early marriage represents the proportion of women aged 20-24 years who were married before age 18; and employment was defined as the percentage of women aged 15-49 years in the community who were currently employed at the time of the survey.

For adults’ contraception use, the collective association of permissive attitudes of adult males towards premarital sex, accepting attitudes of wife-beating, educational achievement, early marriage, and currently working women remained significant in Benin and Nigeria (**Table 4**).

We found similar community-level associations for adolescent peer or adult effects on the demand satisfied with any contraceptive method, although with attenuated effects (**Table 4**). We also found evidence of heterogeneous size effects for these outcomes across countries, with significant collective effects of female accepting attitudes towards wife-beating on the total demand satisfied with any method of adolescent and adult women (Table 4) in Zambia. Positive significant associations of the variation of collective women’s empowerment with the demand satisfied of adult women were found in Niger.

### Assessment of community-level effects

**Figure 3:**
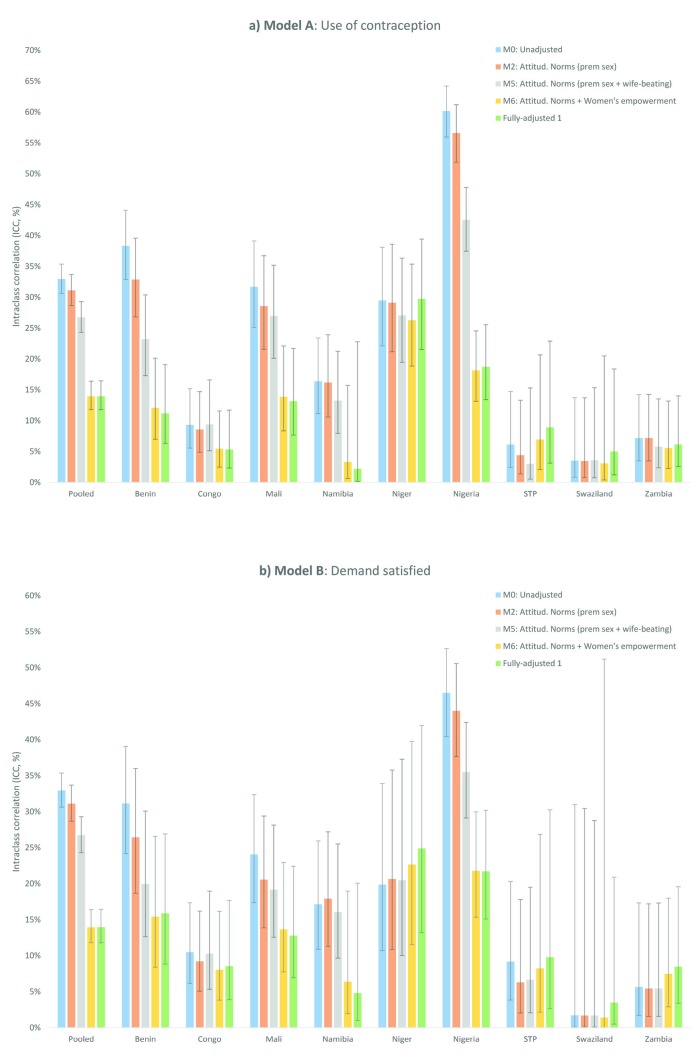
Community-level effects of collective attitudinal norms and indicators of women’s empowerment on the use of (panel a: Model A), and demand satisfied for (panel b: Model B), contraception among young women aged 15-24 years in the pooled sample and by country. STP – Sao Tome and Principe, M0 – Model 0, M2 –Model 2, M5 – Model 5, M6 – Model 6.

In pooled unadjusted multilevel models (M0), we estimated that the community-level random variance (variance (V_2_) = 1.62, 95% CI = 1.45-1.80) represented 33.0% (intra-class correlation (ICC) = 33.0, 95% CI = 30.0-35.4) of the total community-level variation of contraception use. This indicates that community-level differences played an important role in explaining the variation of contraception use at that level. In particular, collective attitudes towards premarital sex reduced the unexplained variance by 4.8% (variance explained (VE) = 4.8), reducing the random variance to 1.54 (V_2_ = 1.54, 95% CI = 1.38-1.72) and the corresponding share of the total attributed variance at that level to 31.9% (ICC = 31.9, 95% CI = 29.5-34.4). After further controlling for individual attitudes (M2), we found that attitudinal norms towards premarital sex explained 8.1% of the variation at that level. Similarly, accepting attitudinal norms towards wife-beating (M4) explained 19.3% of the community-level associated variance with a corresponding reduction in the shared attributed variance to 28.4% (ICC = 28.4, 95% CI = 26.1-30.8). The combined effect of attitudinal norms towards premarital sex and wife-beating explained 25.8% of community-level attributed variance (M5). An additional 41.2% reduction of the community-level associated variance was attributed to the combined effect of educational achievement, early marriage, and work status (M6), reducing the share of the residual variation at that level even further (ICC = 13.9, 95% CI = 11.8-16.4). In a fully-adjusted model (FA1), the addition of age, parity, wealth status, and residency did not contribute any further reduction of the community-level variance (**Figure 3** Panel A; and Table S2 in the **Online Supplementary Document**).

Country-specific analysis revealed marked heterogeneities in the variance attributed to community-level differences in contraceptive use among young women aged 15-24 years, with shares varying from 3.5% (ICC = 3.5, 95% CI = 0.8-13.7) in Swaziland to 38.3% in Benin (ICC = 38.3, 95% CI = 32.9-44.1) and 60.2% in Nigeria (ICC = 60.2, 95% CI = 56.0-64.2) in unadjusted models. Therefore, individual and collective attitudinal norms were likely more meaningful in those countries with larger variance attributed to community-level differences in contraceptive use and significant community-level size effects. For instance, in augmented models for Nigeria (with the largest ICC of 60.2%), individual and collective permissive attitudes towards premarital sex, wife beating, and women’s empowerment jointly explained 85.3% of the total community-level variance (M6) (13.6% [M2], 43.1% [M4], and 34.4% [M6-M5], respectively) (**Figure 3** Panel A; and Table S2 in the **Online Supplementary Document**). In contrast, community-level effects were not meaningful in Sao Tome and Principe, with a low share in the variance attributed to community-level differences in unadjusted models of 6.1% that declined to 3.0% after the combined effect of collective permissive attitudes towards premarital sex and violence, but with no significant effects in the variance explained for collective factors (**Figure 3**) and the corresponding community-level associations (Table S2 in the **Online Supplementary Document**). Similar results were found for the use of contraception among adult women aged 25-49 years (Table S2 in the **Online Supplementary Document**), and for the demand satisfied with any contraceptive method (**Figure 3** Panel B; and Table S3 in the **Online Supplementary Document**).

## DISCUSSION

Despite extensive evidence on the association of gender norms and social influence on contraceptive practice and the total demand for family planning [17-26,51], comprehensive studies on a global scale are challenging due to data limitations specifically on gender norms. Using cross-sectional data from nine SSA countries, this study examined the association between collective attitudes towards premarital sex and wife-beating with women’s contraceptive uptake and demand. In the case of premarital sex, we used the item in DHS regarding whether ‘young women should wait for sex until marriage’, and at the community level we defined permissive attitudes as the proportion of male or female peers (15-24 years) or adults (25-49 years) in the same community who disagreed with that statement. Meanwhile, for women’s acceptance of wife-beating, we used the item ‘It is the respondent’s opinion that a husband is justified in hitting or beating his wife when she refuses to have sex with him’, and collective/contextual level accepting attitudes were defined similarly but considering the opinion of all women aged 15-49 years in the community.

Five salient findings resulted from this study. First, we found high levels of unmet need among women with any demand for family planning (46% in the pooled sample), with significant variations across countries [13] – from 21% in Namibia to 76% in Mali. We found an inverse association between unmet need and the degree of contraception knowledge. Despite that correlation, however, we observed that the vast majority of the people in most countries in our sample have heard of contraception: around 85% in Mali, Niger and Nigeria, and close or equal to 100% in the remaining countries.

Second, we found significant positive associations of collective (peer/adult) permissive attitudes towards premarital sex with the use of, and demand for, contraception for both adolescent and adult women in fully-adjusted models. For adolescent women, a 1 SD increase in the variation of female or male peers with collective permissive attitudes at the community level was associated with 8 to 11% increases in the likelihood of contraceptive use and demand satisfied. Similarly, collective permissive attitudes of adult females or males were associated with increases in the odds of contraceptive use in the range of 4 to 8%. These findings of positive associations of collective influences of peers and/or adults on women’s family planning decisions are in line with previous studies that emphasised the importance of cultural factors, social influence, and contextual effects as important predictors of family planning decision-making of women in SSA [9,16,52]. Ours is the first study to define these relationships quantitatively using proxies for gender norms at the community level. Contraceptive use and demand were also higher among women with higher education, socioeconomic status or parity, or who were unmarried or currently working; these associations are also consistent with previous evidence [53].

Third, collective accepting attitudes towards wife-beating were negatively associated with the use and demand for contraception in our pooled sample for both adolescent and adult women. Both young and adult women were approximately 10% less likely to use contraception or have their demand satisfied when the variation of women’s acceptance of wife-beating increased across communities. These results are in line with recent findings reporting a negative association between contraception use and women’s perceptions of domestic violence in representative countries from West and Central Africa [38].

Fourth, an important contribution of our study is that collective attitudinal norms towards premarital sex and domestic violence jointly explained nearly 26% of the community-level attributed variation of contraceptive use. The association effects for these predictors were attenuated – but remained stable and statistically significant – in the presence of individual and community-level indicators of women’s empowerment, which explained an additional 41% of the community-level attributed variance. The association of women’s empowerment in the variation of contraceptive use was important, but only educational achievement was consistently significant at the community level, also in line with recent findings reporting mixed positive or null results in the complex association of women’s empowerment and family planning practices [54,55].

Fifth, we identified heterogeneous specific and general community-level associations across countries. The relative importance of variation attributed to community-level factors ranged from as low as 3.5% in Swaziland and as much as 60% in Nigeria, indicating that the community-level effects of collective permissive norms are likely more relevant in countries with large general community effects (Benin, Mali, Namibia, Niger, and Nigeria). Significant positive associations of collective permissive attitudes towards premarital sex and domestic violence with contraceptive use were found mainly in separate models for Benin and Nigeria. In both Benin and Nigeria, recent evidence suggests that social and cultural barriers to contraception remain strong, with opposition from partners or social disapproval of sexual activity and contraceptive use being some of the major barriers [20,21,51,56]. Recent evidence also emphasised the importance of contraceptive awareness among adolescents, given the high prevalence of risky sexual behavior (eg, multiple sexual partners) observed in a small region in a rural town in Nigeria [57]. Attitudes towards this kind of behavior may influence family planning practices in specific contexts and requires further examination. In addition, Benin and Nigeria continue to have patriarchal or pronatalist cultures where men have a predominant role in family planning decision-making and they or the family prefer large families. This is consistent with the high percentage of women with no unmet need in our sample (32% in Benin and 34% in Nigeria) [58,59]. The previous findings are particularly relevant for Niger, where the prevalence of no unmet need was the largest among all countries in our sample (50%). Unmet need was large among women with any demand for contraception (58%), and we found counter-intuitive positive associations of accepting attitudes towards wife-beating of women with the use and satisfied demand for contraception for adolescent and adult women in Niger, perhaps attributable to the efforts of women to avoid childbearing in conflictual relationships, as hypothesised in previous studies [60]. Finally, in countries with low contextual attributed variation (<10%, in Congo, Sao Tome and Principe, Swaziland, and Zambia), the evidence and significance of collective attitudinal norms was less relevant in general, although Zambia appears to be an exception. Here we found negative associations of collective accepting attitudes towards wife-beating with contraceptive use and demand satisfied for contraception for adolescents and adult women in Zambia. Previously we reported that collective permissive attitudes toward premarital sex were associated with increased risk for HIV acquisition among young women [27]. Persistence of an extremely patriarchal society and high-levels of gender-based violence that limit the power of women in relationships [61] appear to be relevant to both sets of results. Further research should explore the associations between HIV risk and community-level norms governing contraceptive use and violence against women.

The positive effects of permissive attitudes in our pooled sample, and particularly for Benin and Nigeria may be interpreted in the context of social influence, reflecting the effect of women's perceptions of the views of peers/adults living in the same cluster on their own contraceptive behavior [62]. In addition, community perceptions towards domestic violence can also be interpreted as having detrimental effects on adolescent and adult women’s autonomy to make educated and well-informed family planning decisions. Thus, women may require the approval/disapproval of a reference group (peers/adults) to modify their fertility behaviors or preferences.

### Strengths and limitations

Using the TNS as an interpretive framework to explain the correlation between norms of premarital sex and violence against women with family planning practices, we hypothesise that – granted contextual differences – a norm of premarital sex has a strong influence on people’s sexual practices because the practice is a) interdependent, as the action is coordinated with other(s), even though these others (men) are not necessarily under the same normative influence; b) relatively undetectable in principle, as it involves an intimate act, but potentially highly detectable in the case of pregnancy; c) followed by strong sanctions for non-compliers, several of which are detailed in the relevant literature [63]; and d) proximal to the norm, as the two have a direct relationship [28]. We hypothesised that when attitudes and norms are aligned – when people both personally accept premarital sex and believe others find it acceptable as well – transformative change in women’s family planning practices/preferences may result [64], as suggested by previous evidence showing that sexual behavior has been highly regulated but prone to change [65], although we recognize that our attitudinal norms are only an aggregation of individual attitudes and do not necessary may reflect those beliefs.

This study has a number of limitations. First, our findings relied on observational cross-sectional data that impeded the assessment of causal associations between gender norms and contraceptive practices. Second, because our sample was limited to nine countries in SSA with information on attitudes towards premarital sex for the years between 2005 and 2009, our findings neither can be generalised to the whole region nor assumed to capture the effect of changes in contraceptive practices and attitudes over time. Third, we measured the general contextual effects using the intraclass correlation, but this approximation is based on the assumption of constant individual level random variance as we used a latent model approach for its estimation. Fourth, DHS clusters may not capture social experiences of individuals within their actual communities and, although The DHS Program is committed to maintain high standards of data collection, underlying survey procedures could still affect the assessment of fertility practices and attitudes, particularly in older surveys [66]. Fifth, our measures of gender norms represent a proxy that is based on available data and may not reflect actual norms from communities or capture interpersonal differences in normative change, particularly with regard to approximating social influences of peers and adults. Personal attitudes and norms are different constructs; following work done by others [31,32], we approximated them as ‘collective attitudinal norms’ from peers/adults at the community level.

## CONCLUSIONS

Fertility is declining slowly in SSA and women continue to have more children than they desire. Understanding the reasons behind low contraceptive use is imperative for fertility regulation and avoiding unintended pregnancy. This study investigated the effects of gender norms on family planning practices in nine SSA countries. Our findings provide support of localised approaches to facilitate the dissemination of information on reproductive health in specific countries where the relative importance of community-level variation is meaningful. Our results also provide insights into the importance of addressing barriers related to normative attitudes/behaviors and defusing resistance to contraceptive use by targeting influential groups, as well as for program designs in the future, as they provide detailed information about the main sources of variation of risk factors and potential interventions that can be more relevant in specific contexts/locations. Further research is required to expand the scope of this investigation by examining the linkages between comprehensive collection of data (across time and space) on attitudes, beliefs, social norms, and behaviors with fertility preferences, and reproductive health and practices.

## Additional material

Online Supplementary Document
