# Supplementary material for: Association of collective attitudes and contraceptive practice in nine sub-Saharan African countries
Source: J Glob Health. 2020 Feb 29;10(1):010705. doi: 10.7189/jogh.10.010705 (PMC7101087; doi:10.7189/jogh.10.010705)
Supplement: Online Supplementary Document [file jogh-10-010705-s001.pdf]

Association of Collective Attitudes and Contraceptive Practice in nine sub-Saharan African Countries

Online Supplementary Document

Iván Mejía-Guevara[1,2], Beniamino Cislaghi[3], Ann Weber[4], Emma Hallgren[1], Valerie Meausoone[1], Mark R Cullen[1], Gary L Darmstadt[1,5]

[1] Center for Population Health Sciences, Stanford University School of Medicine, Palo Alto, California, USA
[2] Department of Biology, Stanford University, Palo Alto, California, USA
[3] London School of Hygiene & Tropical Medicine, London, United Kingdom
[4] School of Community Health Sciences, University of Nevada, Reno, Reno, Nevada, USA
[5] Department of Pediatrics, Stanford University School of Medicine, Stanford, California, USA

Appendix S1: Glossary of terms

Here we reproduced basic definitions of need and demand for family planning provided in the Guide to DHS Statistics DHS-7, and illustrated in Figure S1 (1,2):

- Unmet need for contraception. Number of women who are not using a method of contraception but express a desire for spacing or limiting new pregnancies.
- Met need for contraception. Number of women who are using a method of contraception and are not considering to be limiting, do not want more children, are infecund, sterilized, or cannot get pregnant.
- Total demand for contraception. Number of women who have a met need or unmet need.
- Demand satisfied. Number of women who are using any contraceptive method.

The above definitions are based on a sample of women aged 15-49 years, currently married, or sexually active unmarried women—including women who are not currently married or in consensual union (single, divorced, widowed, and separated) and who had sexual intercourse within the last 30 days. For the demand satisfied, the definition additionally considers women who have either unmet or met need for family planning.

Appendix S2: Multilevel Analysis

We performed a 2-level multilevel logistic analysis to estimate cross-level effects between use of, and demand for, contraception of adolescent (aged 15-24 years)/adult(aged 25-49 years) women, and collective attitudes of peers (15-24) or adults(25-49) living in the same community, as well as for the estimation of random effects at each hierarchical level: with individual responses of woman i (level 1), nested within community j (level 2). Multilevel logistic models are more efficient than traditional logistic regression models because they were designed to deal with variation at different levels (3) and they naturally account for the two-stage cluster sampling design of DHS surveys (4).

The model specification of the multilevel approach was as follows:

$$y_{ij} = \beta_0 + B_1 \cdot \boldsymbol{X_{1j}} + B_2 \cdot \boldsymbol{X_{2i}} + \mu_{0j} + e_{ij}, \quad \text{(S1)}$$

Formatted: Justified

Formatted: Justified

where y_{ij} represents the individual level outcome for women *i* in the community *j*; $\boldsymbol{X_{12j}}, \boldsymbol{X_{21i}}$ are vectors of community and individual level predictors, respectively; and the last two terms stand for random effects at the community (μ_{0j}) and individual (e_{ij}) level, respectively, and assuming they are independent and normally distributed random variables.

Formatted: Justified

To compute general contextual effects, we used the intraclass-correlation to measure the proportion of the total variance that is attributed to the community level, providing a good approximation of the variation of the outcome across communities (5), as follows:

$$ICC = \frac{\sigma_\mu^2}{\sigma_\mu^2 + \sigma_e^2} * 100, \qquad \text{(S2)}$$

where σ_μ^2, stands for random variance at the community level (denoted as V_2 in Tables S2 and S3), and σ_e^2 is the variance at the individual level. However, in multilevel logistic models, σ_e^2 cannot be estimated directly, as the variance of a binomial distribution is determined by the mean and the between-cluster variance is defined in a different scale rather than the binary outcome scale and requires alternative methods for its estimation. In this research we used a latent model approach, which assumes that the binary outcome variable arises as a dichotomization of a continuous latent variable that follows a logistic distribution and converges to the constant value of $\frac{\pi^2}{3}$ (5).

Formatted: Justified

To estimate the variance explained by a set of predictors at the contextual level, e.g. $\boldsymbol{X_{1j}}$, we first estimate a reduced model that does not include $\boldsymbol{X_{1j}}$ [using (1)], and compute the resulting variance at the community level ($\hat{\sigma}_{\mu,r}^2$, where *r* stands for reduced model). We then estimate an augmented model adding $\boldsymbol{X_{1j}}$ back to the model specification and save the resulting contextual-level variance ($\hat{\sigma}_{\mu,a}^2$, where *a* stands for augmented model). The percentage variance explained at the community level (VE_μ) is then calculated as follows (6,7):

$$VE_\mu = \frac{(\hat{\sigma}_{\mu,a}^2 - \hat{\sigma}_{\mu,r}^2)}{\hat{\sigma}_{\mu,r}^2} * 100. \qquad \text{(S3)}$$

For this paper, we always estimated the VE relative to an unadjusted or null model that did not include any covariate or risk factor, only random effects [and country fixed-effects in pooled models] (i.e, $\hat{\sigma}_{\mu,r}^2$ resulted from the unadjusted model). We applied this procedure using alternative model specifications to estimate the proportion of the variance explained by individual and collective attitudinal norms, indicators of women's autonomy, and other covariates (as defined by models M1-M6, FA1, and FA2 in the Figure 1 of the main text, and reported in Tables S2 and S3).

Formatted: Justified

References S1

1. Bradley SEK, Croft TN, Fishel JD, Westoff CF. Revising Unmet Need for Family Planning. Calverton, Maryland, USA: ICF International; 2012. Report No.: DHS Analytical Studies No. 25.

2. DHS Program. Need and Demand for Family Planning [Internet]. 2019. (Guide to DHS Statistics DHS-7.). Available from: https://dhsprogram.com/Data/Guide-to-DHS-Statistics/index.htm#t=Need_and_Demand_for_Family_Planning.htm

3. Goldstein H. Multilevel Statistical Models. 4th ed. Wiley; 2010.

4. Heeringa SG, West BT, Berglung PA. Applied Survey Data Analysis. Second Edition. Boca Raton, FL: CRD Press; 2017.

5. Austin PC, Merlo J. Intermediate and advanced topics in multilevel logistic regression analysis. Statistics in Medicine. 2017 Sep 10;36(20):3257–77.

6. Mejía-Guevara I, Krishna A, Corsi DJ, Subramanian SV. Individual and Ecological Variation in Child Undernutrition in India: A Multilevel Analysis. Journal of South Asian Development. 2015 Aug 1;10(2):168–98.

7. Mejía-Guevara I, Corsi DJ, Perkins JM, Kim R, Subramanian SV. Variation in Anthropometric Status and Growth Failure in Low- and Middle-Income Countries. Pediatrics. 2018 Mar 1;141(3):e20172183.

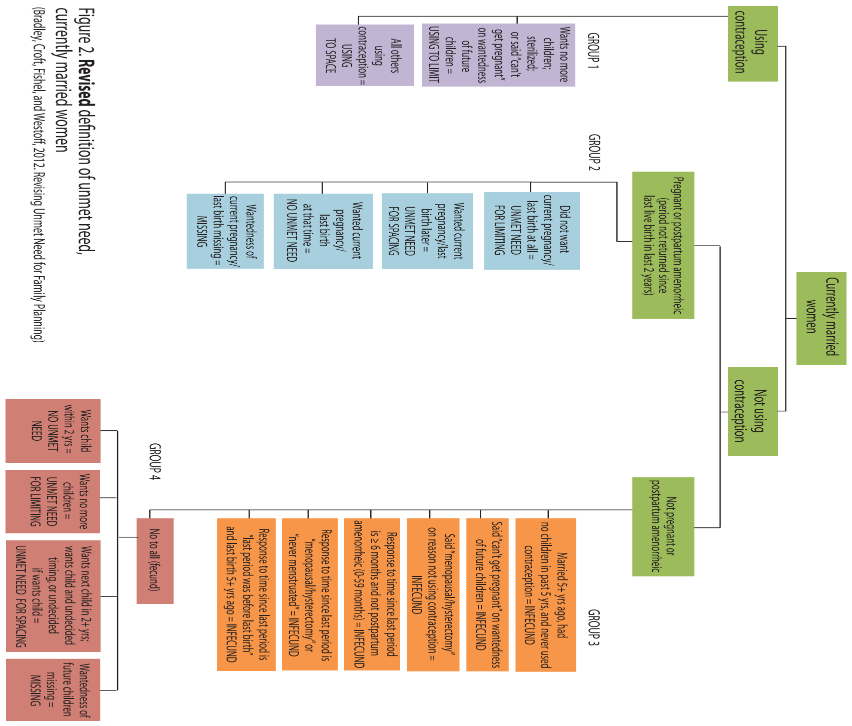

Figure S1. Standard definition of unmet need for currently married women.
Source: Taken from Bradley et al. (2012) (1)

Table S1. Model B: Association of collective (peer and adult) permissive attitudes towards premarital sex and acceptance of wife-beating, and the individual demand satisfied for contraception using logistic two-level multilevel random intercept models for adolescent (aged 15-24 years) and adult (aged ~~15-24~~25-49 years) women in a pooled sample of nine SSA countries.

Outcome: Demand satisfied	Adolescent women (aged 15-24 years)								Adult women (aged 25-49 years)
	M1: collective attitudes towards premarital sex	M2: M1 + individual attitudes towards premarital	M3: collective attitudes towards wife-beating	M4: M3 + individual attitudes towards wife-beating	M5: M2 + M4	M6: M5 + women's empowerment	FA1: M6 + individual level covariates (peers'	FA2: M6 + individual level covariates (adults'	FA2: M6 + individual level covariates (adults' attitudes in M1)
**Community-level variables [OR per 1 SD increase (95% CI)]**									
**Collective attitudinal norms**									
Acceptance of premarital sex (1 SD)									
Female peer	1.15 (1.08 - 1.22)	1.10 (1.03 - 1.17)			1.07 (1.00 - 1.14)	1.06 (0.99 - 1.13)	1.06 (0.99 - 1.13)		
Male peer	1.06 (1.00 - 1.13)	1.07 (1.01 - 1.14)			1.07 (1.01 - 1.14)	1.06 (1.00 - 1.13)	1.07 (1.01 - 1.13)		
Female adult								1.06 (1.00 - 1.12)	1.04 (0.99 - 1.08)
Male adult								1.02 (0.97 - 1.08)	1.04 (0.99 - 1.09)
Acceptance of wife-beating (1 SD)			0.63 (0.59 - 0.67)	0.66 (0.62 - 0.70)	0.67 (0.62 - 0.71)	0.88 (0.82 - 0.94)	0.89 (0.83 - 0.95)	0.89 (0.83 - 0.96)	0.86 (0.81 - 0.90)
**Women's empowerment***									
Secondary/higher school completion (1 SD)						1.33 (1.23 - 1.43)	1.16 (1.06 - 1.27)	1.17 (1.07 - 1.27)	1.24 (1.16 - 1.32)
Early marriage (1 SD)						0.97 (0.91 - 1.03)	1.01 (0.95 - 1.08)	1.02 (0.95 - 1.08)	0.95 (0.91 - 0.99)
Currently working						1.02 (0.95 - 1.09)	1.02 (0.95 - 1.09)	1.02 (0.95 - 1.09)	1.1 (1.05 - 1.16)

* Educational achievement was defined as the proportion of women aged 15-49 years who completed secondary or higher education; early marriage represents the proportion of women aged 20-24 years who were married before age 18; and employment was defined as the percentage of women aged 15-49 years in the community who were currently employed at the time of the survey.

Table S1. Continued

Outcome: Demand satisfied	Adolescent women (aged 15-24 years)								Adult women (aged 25-49 years)
	**M1:** collective attitudes towards premarital sex	**M2:** M1 + individual attitudes towards premarital sex	**M3:** collective attitudes towards wife-beating	**M4:** M3 + individual attitudes towards wife-beating	**M5:** M2 + M4	**M6:** M5 + women's empowerment	**FA1:** M6 + individual level covariates (peers' attitudes in M1)	**FA2:** M6 + individual level covariates (adults' attitudes in M1)	**FA2:** M6 + individual level covariates (adults' attitudes in M1)
**Individual-level variables [OR (95% CI)]**									
Individual permissive attitudes towards premarital sex		1.29			1.28	1.20	1.23	1.23	0.97
		(1.11 - 1.51)			(1.10 - 1.50)	(1.02 - 1.41)	(1.04 - 1.45)	(1.06 - 1.43)	(0.85 - 1.10)
Individual accepting attitudes towards wife-beating				0.80	0.79	0.88	0.88	0.91	1.01
				(0.70 - 0.91)	(0.69 - 0.91)	(0.76 - 1.02)	(0.76 - 1.02)	(0.78 - 1.05)	(0.92 - 1.11)
Level of education (None = 1)									
Primary						1.76	1.80	1.79	1.68
						(1.48 - 2.09)	(1.50 - 2.15)	(1.51 - 2.12)	(1.52 - 1.87)
Secondary						2.52	2.38	2.41	2.14
						(2.08 - 3.04)	(1.95 - 2.90)	(1.99 - 2.92)	(1.89 - 2.43)
Higher						5.12	3.25	3.35	3.36
						(3.54 - 7.40)	(2.19 - 4.80)	(2.29 - 4.92)	(2.71 - 4.15)
Marital status									
Never in union						2.19	2.42	2.46	2.21
						(1.91 - 2.51)	(2.05 - 2.85)	(2.09 - 2.89)	(1.77 - 2.76)
Formerly in union						1.51	1.53	1.49	1.88
						(1.07 - 2.13)	(1.07 - 2.19)	(1.06 - 2.11)	(1.55 - 2.27)
Currently working						1.34	1.24	1.20	1.28
						(1.18 - 1.52)	(1.09 - 1.41)	(1.06 - 1.36)	(1.16 - 1.41)
Age							1.14	1.14	0.99
							(1.11 - 1.17)	(1.11 - 1.17)	(0.98 - 1.00)
Number of live births (No children = 1)									
1							0.72	0.71	0.85
							(0.61 - 0.85)	(0.60 - 0.84)	(0.62 - 1.16)
2							0.82	0.81	0.75
							(0.66 - 1.01)	(0.66 - 1.00)	(0.55 - 1.01)
3							0.78	0.77	0.79
							(0.59 - 1.02)	(0.59 - 1.01)	(0.58 - 1.06)
4 or more							0.54	0.53	0.75
							(0.38 - 0.77)	(0.37 - 0.74)	(0.55 - 1.01)
Wealth quintile (Q1 = 1)									
Q2							1.05	1.07	1.11
							(0.86 - 1.28)	(0.88 - 1.29)	(0.98 - 1.26)
Q3							0.99	1.04	1.26
							(0.81 - 1.21)	(0.86 - 1.25)	(1.11 - 1.43)
Q4							1.27	1.29	1.71
							(1.02 - 1.57)	(1.05 - 1.58)	(1.49 - 1.96)
Q5							1.60	1.67	2.12
							(1.25 - 2.06)	(1.31 - 2.12)	(1.80 - 2.49)
Residency (Rural = 1)									
Urban							1.00	0.98	0.95
							(0.86 - 1.17)	(0.84 - 1.14)	(0.85 - 1.06)
Observations	12,622	12,028	13,540	13,047	11,630	10,408	10,408	11,072	22,765
Number of groups	3,124	3,102	3,432	3,405	3,080	2,678	2,678	2,914	3,201

For adolescent women, models are presented adjusting for peers' collective attitudes (FA1 model) and adults' collective attitudes (FA2 model). We additionally controlled for country fixed-effects in all models.
M1-M6 –Models 1 to 6, FA1 –Fully-adjusted Model 1, FA2 –Fully-adjusted Model 2, OR –Odds ratio, SD –Standard deviation, CI –Confidence interval.

Table S2. Random variation at the community level (V_2), intraclass-correlation (ICC), and variance explained (VE)* of use of (Model A), and demand satisfied for (Model B), contraception using logistic two-level multilevel -random intercept models for adolescent (aged 15-24 years) women in pooled samples and by country.

Country	Indicator	M0: unadjusted	M1: collective attitudes towards premarital sex	M2: M1 + individual attitudes towards premarital sex	M3: M2 + collective attitudes towards wife-beating	M4: M3 + individual attitudes towards wife-beating	M5: M2 + M4	M6: M5 + women's empowerment	FA1: M6 + individual level covariates (peers' attitudes in	FA2: M6 + individual level covariates (adults' attitudes in
**Model A: Use of contraception**										
Pooled	V_2	1.62 (1.45-1.8)	1.54 (1.38-1.72)	1.49 (1.32-1.67)	1.3 (1.16-1.45)	1.31 (1.16-1.47)	1.2 (1.06-1.36)	0.53 (0.44-0.65)	0.53 (0.44-0.65)	0.56 (0.46-0.67)
	ICC	33 (30.6-35.4)	31.9 (29.5-34.4)	31.1 (28.7-33.7)	28.3 (26.1-30.6)	28.4 (26.1-30.8)	26.7 (24.3-29.3)	13.9 (11.8-16.4)	14 (11.8-16.4)	14.5 (12.3-16.9)
	VE		4.8	8.1	19.7	19.3	25.8	67	67	65.6
Benin	V_2	2 (1.6-2.6)	1.6 (1.2-2.2)	1.6 (1.2-2.2)	1.3 (1-1.8)	1.3 (1-1.8)	1 (0.7-1.4)	0.5 (0.2-0.8)	0.4 (0.2-0.8)	0.5 (0.3-0.9)
	ICC	38.3 (32.9-44.1)	33.1 (27.2-39.6)	32.9 (26.8-39.6)	28.9 (23.5-35)	28.7 (23.2-34.9)	23.2 (17.3-30.4)	12.1 (7-20.1)	11.2 (6.3-19.1)	13.4 (8.3-20.8)
	VE		20.2	21.2	34.5	35.2	51.4	77.9	79.7	75.1
Congo	V_2	0.3 (0.2-0.6)	0.3 (0.2-0.6)	0.3 (0.2-0.6)	0.3 (0.2-0.6)	0.4 (0.2-0.7)	0.3 (0.2-0.7)	0.2 (0.1-0.4)	0.2 (0.1-0.4)	0.2 (0.1-0.4)
	ICC	9.3 (5.6-15.2)	9.5 (5.7-15.4)	8.6 (4.9-14.7)	9.2 (5.4-15.2)	10 (5.7-16.9)	9.4 (5.1-16.6)	5.5 (2.5-11.6)	5.3 (2.3-11.7)	4.9 (2.1-11.3)
	VE		-2.2	8.5	1.3	-8.1	-1	43.9	45.2	49.5
Mali	V_2	1.5 (1.1-2.1)	1.5 (1-2.1)	1.3 (0.9-1.9)	1.5 (1-2)	1.4 (1-2)	1.2 (0.8-1.8)	0.5 (0.3-0.9)	0.5 (0.3-0.9)	0.6 (0.4-1.1)
	ICC	31.7 (25.1-39.1)	30.8 (23.8-38.7)	28.6 (21.6-36.7)	30.8 (24.1-38.3)	30.2 (23.6-37.7)	27 (20.1-35.2)	13.8 (8.3-22.1)	13.2 (7.7-21.7)	16.3 (10.4-24.7)
	VE		4.3	13.9	4.2	6.8	20.4	65.4	67.3	57.9
Namibia	V_2	0.6 (0.4-1)	0.7 (0.4-1.1)	0.6 (0.4-1)	0.4 (0.3-0.8)	0.5 (0.3-0.8)	0.5 (0.3-0.9)	0.1 (0-0.6)	0.1 (0-1)	0 (0-1.5)
	ICC	16.4 (11.2-23.4)	17.2 (11.6-24.8)	16.2 (10.6-23.9)	12 (7.3-19.1)	13 (8-20.4)	13.2 (7.9-21.3)	3.3 (0.6-15.7)	2.2 (0.2-22.8)	1.5 (0-31.8)
	VE		-6	1.5	30.5	23.8	22.1	82.4	88.6	92.4
Niger	V_2	1.4 (0.9-2)	1.3 (0.9-2)	1.4 (0.9-2.1)	1.2 (0.8-1.8)	1.2 (0.8-1.8)	1.2 (0.8-1.9)	1.2 (0.8-1.8)	1.4 (0.9-2.1)	1.4 (0.9-2.1)
	ICC	29.5 (22.1-38.1)	28.5 (20.9-37.7)	29.1 (21.2-38.6)	27.5 (20.5-35.9)	27.2 (20.2-35.7)	27.1 (19.4-36.3)	26.3 (18.8-35.4)	29.7 (21.5-39.4)	29.7 (21.9-38.9)
	VE		4.5	1.8	9.2	10.5	11.3	14.8	-1.1	-1.2
Nigeria	V_2	5 (4.2-5.9)	4.4 (3.6-5.3)	4.3 (3.5-5.2)	2.8 (2.3-3.4)	2.8 (2.3-3.4)	2.4 (2-3)	0.7 (0.5-1.1)	0.8 (0.5-1.1)	0.8 (0.6-1.2)
	ICC	60.2 (56-64.2)	57.1 (52.5-61.6)	56.6 (51.9-61.2)	45.9 (41.1-50.7)	46.2 (41.3-51.2)	42.5 (37.5-47.8)	18.2 (13.1-24.5)	18.7 (13.4-25.5)	19.6 (14.3-26.2)
	VE		11.8	13.6	43.8	43.1	50.9	85.3	84.7	83.9
Sao Tome and Principe	V_2	0.2 (0.1-0.6)	0.2 (0.1-0.5)	0.2 (0-0.5)	0.2 (0.1-0.6)	0.2 (0.1-0.7)	0.1 (0-0.6)	0.2 (0.1-0.9)	0.3 (0.1-1)	0.3 (0.1-1)
	ICC	6.1 (2.4-14.7)	5.5 (2.1-13.8)	4.4 (1.4-13.3)	5.9 (2.3-14.4)	6 (2-16.8)	3 (0.5-15.3)	6.9 (2.1-20.7)	8.9 (3.1-22.9)	8.9 (3.1-23.1)
	VE		10.7	29.4	3.9	2.6	52.6	-14.1	-49.7	-50.2
Swaziland	V_2	0.1 (0-0.5)	0.1 (0-0.5)	0.1 (0-0.5)	0.1 (0-0.6)	0.1 (0-0.6)	0.1 (0-0.6)	0.1 (0-0.8)	0.2 (0-0.7)	0.2 (0-0.7)
	ICC	3.5 (0.8-13.7)	3.6 (0.9-13.5)	3.5 (0.8-13.7)	2.9 (0.5-14.6)	3.5 (0.7-15.6)	3.6 (0.8-15.3)	3.1 (0.4-20.5)	5 (1.2-18.4)	4.5 (1-18.2)
	VE		-1.1	1.9	18.5	1.9	-1.5	12.8	-44.5	-30.1
Zambia	V_2	0.3 (0.1-0.5)	0.3 (0.1-0.5)	0.3 (0.1-0.5)	0.2 (0.1-0.5)	0.2 (0.1-0.5)	0.2 (0.1-0.5)	0.2 (0.1-0.5)	0.2 (0.1-0.5)	0.2 (0.1-0.5)
	ICC	7.2 (3.5-14.2)	7.2 (3.5-14.3)	7.2 (3.5-14.2)	5.9 (2.5-13.5)	5.8 (2.4-13.5)	5.8 (2.4-13.5)	5.6 (2.2-13.2)	6.2 (2.6-14)	6 (2.5-13.8)
	VE		-0.9	0	18.6	19.8	20.6	23.7	15	17.2

* Variance explained was estimated using the unadjusted model as reference, then indicating the percentage of variance explained by the risk factors or predictors in each separate model. ICC and VE are reported as %.

Table S2. Continued

Country	Indicator	M0: unadjusted	M1: collective attitudes towards premarital sex	M2: M1 + individual attitudes towards premarital sex	M3: M2 + collective attitudes towards wife-beating	M4: M3 + individual attitudes towards wife-beating	M5: M2 + M4	M6: M5 + women's empowerment	FA1: M6 + individual level covariates (peers' attitudes in M1)	FA2: M6 + individual level covariates (adults' attitudes in M1)
**Model B: Demand satisfied with any method**										
Pooled	V_2	1.04 (0.89-1.21)	1.01 (0.86-1.19)	0.96 (0.82-1.14)	0.89 (0.76-1.04)	0.92 (0.78-1.08)	0.85 (0.71-1.02)	0.54 (0.43-0.68)	0.53 (0.42-0.67)	0.54 (0.43-0.67)
	ICC	33 (30.6-35.4)	31.9 (29.5-34.4)	31.1 (28.7-33.7)	28.3 (26.1-30.6)	28.4 (26.1-30.8)	26.7 (24.3-29.3)	13.9 (11.8-16.4)	14 (11.8-16.4)	14.5 (12.3-16.9)
	VE		2.5	7.2	14.5	11.7	17.7	47.8	48.5	48
Benin	V_2	1.5 (1-2.1)	1.2 (0.8-1.9)	1.2 (0.8-1.9)	1 (0.7-1.6)	1 (0.7-1.6)	0.8 (0.5-1.4)	0.6 (0.3-1.2)	0.6 (0.3-1.2)	0.7 (0.4-1.3)
	ICC	31.1 (24.2-39.1)	27.3 (19.8-36.3)	26.4 (18.7-36)	23.6 (16.9-32)	24.2 (17.3-32.7)	20 (12.6-30.1)	15.4 (8.4-26.6)	15.9 (8.8-26.9)	17.7 (10.8-27.8)
	VE	0	16.9	20.5	31.6	29.5	44.9	59.7	58.2	52.3
Congo	V_2	0.4 (0.2-0.7)	0.4 (0.2-0.7)	0.3 (0.2-0.6)	0.4 (0.2-0.7)	0.4 (0.2-0.8)	0.4 (0.2-0.8)	0.3 (0.1-0.6)	0.3 (0.1-0.7)	0.3 (0.1-0.7)
	ICC	10.5 (6.1-17.3)	10.7 (6.3-17.7)	9.2 (5.1-16.2)	10.4 (6.1-17.4)	11.6 (6.5-19.9)	10.3 (5.3-19)	8.1 (3.8-16.2)	8.5 (3.9-17.7)	8.6 (4.1-17.2)
	VE	0	-2.7	13.3	0.4	-12	2	25.3	20.3	20
Mali	V_2	1 (0.7-1.6)	1 (0.7-1.6)	0.9 (0.5-1.4)	1 (0.7-1.5)	1 (0.6-1.5)	0.8 (0.5-1.3)	0.5 (0.3-1)	0.5 (0.2-1)	0.5 (0.3-1)
	ICC	24.1 (17.4-32.4)	24.1 (17-32.9)	20.6 (13.9-29.4)	23.5 (16.8-31.8)	22.9 (16.1-31.4)	19.2 (12.5-28.1)	13.6 (7.7-22.9)	12.8 (6.9-22.4)	14.2 (8.2-23.4)
	VE	0	0	18.3	3.4	6.4	25.3	50.1	53.7	47.8
Namibia	V_2	0.7 (0.4-1.2)	0.8 (0.4-1.3)	0.7 (0.4-1.2)	0.5 (0.3-1)	0.6 (0.3-1.1)	0.6 (0.4-1.1)	0.2 (0.1-0.8)	0.2 (0-0.8)	0.1 (0-0.9)
	ICC	17.1 (10.9-25.9)	18.6 (11.9-27.7)	17.9 (11.3-27.2)	13.8 (8.1-22.6)	15.5 (9.4-24.5)	16.1 (9.7-25.5)	6.4 (1.9-19)	4.8 (1-20.1)	4.2 (0.7-21.2)
	VE	0	-10.2	-5.5	22.4	11.5	7.5	67	75.6	78.7
Niger	V_2	0.8 (0.4-1.7)	0.8 (0.4-1.7)	0.9 (0.4-1.8)	0.7 (0.3-1.6)	0.7 (0.3-1.7)	0.8 (0.4-2)	1 (0.4-2.2)	1.1 (0.5-2.4)	1.1 (0.5-2.2)
	ICC	19.9 (10.7-33.9)	19.6 (10.1-34.6)	20.7 (10.8-35.8)	18.1 (9-33.1)	18.5 (9.2-33.9)	20.5 (10-37.3)	22.7 (11.5-39.7)	24.9 (13.2-42)	24.6 (13.5-40.6)
	VE	0	2	-5	11	8.2	-3.8	-18.2	-33.7	-31.5
Nigeria	V_2	2.9 (2.2-3.7)	2.6 (2-3.4)	2.6 (2-3.4)	1.9 (1.4-2.5)	2 (1.5-2.6)	1.8 (1.4-2.4)	0.9 (0.6-1.4)	0.9 (0.6-1.4)	0.9 (0.6-1.5)
	ICC	46.5 (40.4-52.7)	44.1 (37.9-50.6)	44 (37.7-50.6)	36.6 (30.5-43.1)	37.4 (31-44.2)	35.5 (29.1-42.4)	21.8 (15.3-30)	21.7 (15.1-30.2)	22.4 (15.8-30.8)
	VE	0	9.1	9.6	33.7	31.3	36.7	68	68.1	66.8
Sao Tome and Principe	V_2	0.3 (0.1-0.8)	0.3 (0.1-0.7)	0.2 (0.1-0.7)	0.3 (0.1-0.8)	0.4 (0.2-0.9)	0.2 (0.1-0.8)	0.3 (0.1-1.2)	0.4 (0.1-1.4)	0.3 (0.1-1.4)
	ICC	9.2 (3.8-20.3)	7.9 (3.2-18.1)	6.3 (2-17.8)	9.4 (4-20.5)	10.4 (4.7-21.7)	6.7 (2.1-19.5)	8.2 (2.2-26.8)	9.8 (2.6-30.3)	9.5 (2.5-30.5)
	VE	0	15.1	33.5	-3.4	-15.2	29.2	10.9	-7.7	-4.1
Swaziland	V_2	0.1 (0-1.5)	0.1 (0-1.3)	0.1 (0-1.4)	0 (0-4.1)	0.1 (0-1.7)	0.1 (0-1.3)	0 (0-3.4)	0.1 (0-0.9)	0.1 (0-0.9)
	ICC	1.7 (0.1-31)	1.7 (0.1-28.9)	1.7 (0.1-30.4)	1.1 (0-55.7)	1.6 (0-33.6)	1.7 (0.1-28.8)	1.4 (0-51.2)	3.5 (0.5-20.9)	3 (0.3-22.2)
	VE	0	-2.2	1.2	36	9.3	0.7	18.4	-107.5	-76
Zambia	V_2	0.2 (0.1-0.7)	0.2 (0.1-0.7)	0.2 (0.1-0.7)	0.2 (0-0.7)	0.2 (0.1-0.7)	0.2 (0.1-0.7)	0.3 (0.1-0.7)	0.3 (0.1-0.8)	0.3 (0.1-0.8)
	ICC	5.7 (1.7-17.3)	5.6 (1.7-17.2)	5.4 (1.6-17.2)	4.9 (1.3-16.7)	5.9 (1.8-17.6)	5.5 (1.6-17.3)	7.5 (2.9-18)	8.5 (3.4-19.6)	8.6 (3.5-19.9)
	VE	0	1.4	4.6	14.7	-4.3	4.2	-34.4	-53.7	-57.1

V_2 –Random variance at the community level, ICC –Intra-class correlation, VE –Variance explained. M1-M6 – Models 1 to 6, FA1 –Fully-adjusted model 1, FA2 –Fully-adjusted model 2.

Fully-adjusted 1 (FA1) models were adjusted using peers' collective attitudes, while for FA2 adults' collective attitudes were used for the adjustment. ICC and VE are reported as %.

Table S3. Random variation at the community level (V_2), intraclass-correlation (ICC), and variance explained (VE)* of use of (Model A), and demand satisfied for (Model B), contraception using logistic two-level multilevel random intercept models for adult (25-49 years) women in pooled samples and by country.

Country	Indicator	M0: unadjusted	M2: collective and individual attitudes towards premarital sex	M5: M2 + collective and individual attitudes towards wife-beating	M6: M5 + women's empowerment	FA2: individual level covariates (adults' attitudes in M1)
**Model A: Use of contraception**						
Pooled	V_2	1.09 (1.01-1.19)	1.06 (0.97-1.15)	0.86 (0.79-0.94)	0.56 (0.5-0.63)	0.56 (0.49-0.63)
	ICC	24.9 (23.4-26.5)	24.3 (22.8-25.9)	20.7 (19.3-22.2)	14.6 (13.1-16.2)	14.5 (13-16.1)
	VE		3.2	21.3	48.7	49
Benin	V_2	1.1 (1-1.4)	1 (0.8-1.2)	0.7 (0.6-0.9)	0.5 (0.4-0.7)	0.5 (0.4-0.7)
	ICC	25.8 (22.5-29.4)	23.3 (20-27)	18.5 (15.4-22)	14.1 (10.8-18.1)	13.8 (10.5-17.9)
	VE	0	12.5	34.9	53	54.1
Congo	V_2	0.1 (0.1-0.3)	0.2 (0.1-0.3)	0.2 (0.1-0.3)	0.1 (0.1-0.3)	0.2 (0.1-0.3)
	ICC	4.3 (2.3-7.9)	4.9 (2.8-8.5)	5 (2.9-8.5)	4.1 (2-7.9)	4.5 (2.5-8.3)
	VE	0	-16	-18.9	4.5	-7.2
Mali	V_2	1.1 (0.8-1.5)	1 (0.7-1.4)	1 (0.7-1.4)	0.5 (0.3-0.8)	0.5 (0.3-0.8)
	ICC	24.9 (19.5-31.1)	23.3 (18-29.7)	23 (17.7-29.3)	13 (8.4-19.5)	12.4 (7.7-19.2)
	VE	0	8	9.6	55	57.3
Namibia	V_2	0.5 (0.3-0.6)	0.4 (0.3-0.6)	0.2 (0.1-0.4)	0.1 (0-0.4)	0 (0-0)
	ICC	12.2 (9-16.4)	11.1 (7.7-15.8)	6.4 (3.7-10.7)	2.3 (0.5-10.3)	0 (0-0)
	VE	0	10	51	83.4	100
Niger	V_2	1.9 (1.4-2.4)	1.8 (1.4-2.3)	1.7 (1.3-2.2)	1.1 (0.8-1.5)	1.1 (0.8-1.4)
	ICC	36 (30.5-42)	35.7 (30.1-41.6)	33.7 (28.1-39.8)	24.7 (19.5-30.7)	24.3 (19.2-30.3)
	VE	0	1.6	9.9	41.9	42.9
Nigeria	V_2	2.4 (2.1-2.8)	2.2 (1.9-2.5)	1.2 (1-1.4)	0.5 (0.3-0.6)	0.5 (0.4-0.6)
	ICC	42.4 (39.2-45.7)	40.2 (37-43.4)	26.3 (23.2-29.7)	12.2 (9.6-15.4)	12.6 (9.9-16)
	VE	0	9	51.6	81.1	80.4
Sao Tome and Principe	V_2	0.2 (0.1-0.5)	0.2 (0.1-0.4)	0.2 (0.1-0.5)	0.2 (0.1-0.5)	0.2 (0.1-0.5)
	ICC	6.2 (3-12.4)	5 (2.2-10.8)	6 (2.8-12.4)	4.9 (1.9-12.5)	4.8 (1.8-12.4)
	VE	0	20.6	3.9	21.6	24.3
Swaziland	V_2	0.2 (0.1-0.4)	0.2 (0.1-0.4)	0.2 (0.1-0.4)	0.2 (0.1-0.5)	0.2 (0.1-0.6)
	ICC	6.3 (3.5-11.3)	6.1 (3.3-11.2)	5.4 (2.6-10.9)	5.8 (2.3-13.9)	6.2 (2.3-15.9)
	VE	0	3.4	15.7	8.7	1.9
Zambia	V_2	0.5 (0.4-0.7)	0.5 (0.3-0.7)	0.3 (0.2-0.5)	0.3 (0.2-0.4)	0.3 (0.2-0.5)
	ICC	13.7 (10.1-18.5)	12.8 (9.2-17.4)	9 (6.1-13.2)	7.6 (4.8-12)	8 (5-12.6)
	VE	0	8.1	37.7	48.1	45.3

FA2 models were adjusted using adults' collective attitudes.

* Variance explained was estimated using the unadjusted model as reference, then indicating the percentage of variance explained by the risk factors or predictors in each separate model. ICC and VE are reported as %.

Table S3. Continued

Country	Indicator	M0: unadjusted	M2: collective and individual attitudes towards premarital sex	M5: M2 + collective and individual attitudes towards wife-beating	M6: M5 + women's empowerment	FA2: M6 + individual level covariates (adults' attitudes in M1)
**Model B: Demand satisfied with any method**						
Pooled	V_2	1.04 (0.94-1.15)	1.02 (0.92-1.13)	0.82 (0.74-0.92)	0.59 (0.51-0.68)	0.59 (0.51-0.68)
	ICC	24.9 (23.4-26.5)	24.3 (22.8-25.9)	20.7 (19.3-22.2)	14.6 (13.1-16.2)	14.5 (13-16.1)
	VE		1.9	20.8	43	43.6
Benin	V_2	1.1 (0.8-1.3)	1 (0.8-1.2)	0.8 (0.6-1)	0.6 (0.4-0.8)	0.6 (0.4-0.8)
	ICC	24.5 (20.5-29)	22.8 (18.9-27.4)	18.7 (15.1-23.1)	15.5 (11.8-20.2)	15.3 (11.5-20)
	VE	0	8.8	28.9	43.4	44.4
Congo	V_2	0.2 (0.1-0.4)	0.2 (0.1-0.4)	0.2 (0.1-0.4)	0.2 (0.1-0.5)	0.2 (0.1-0.5)
	ICC	4.7 (2-10.5)	5.1 (2.2-11.3)	5.6 (2.6-11.5)	6 (2.8-12.3)	5.7 (2.6-12.1)
	VE	0	-10.5	-20.5	-30.5	-24.2
Mali	V_2	0.9 (0.7-1.3)	0.9 (0.6-1.2)	0.8 (0.6-1.2)	0.5 (0.3-0.7)	0.4 (0.3-0.7)
	ICC	21.7 (16.6-27.8)	20.8 (15.7-27)	20.4 (15.2-26.7)	12.1 (8.2-17.5)	11.9 (8-17.3)
	VE	0	5.1	7.4	50.1	51.3
Namibia	V_2	0.7 (0.5-0.9)	0.7 (0.5-1)	0.4 (0.3-0.7)	0.1 (0-0.7)	0 (0-29.3)
	ICC	16.7 (12.4-22.1)	16.6 (12-22.5)	11.7 (7.9-17)	4 (0.8-17.3)	0.8 (0-89.9)
	VE	0	0.6	34	79.1	95.8
Niger	V_2	1.2 (0.8-1.8)	1.2 (0.9-1.8)	1.2 (0.8-1.7)	0.9 (0.6-1.4)	1 (0.6-1.5)
	ICC	26.8 (20.2-34.7)	27.4 (20.6-35.4)	26.3 (19.4-34.6)	22 (15.4-30.4)	22.5 (15.9-31)
	VE	0	-2.7	2.8	23.1	20.7
Nigeria	V_2	2.3 (2-2.8)	2.2 (1.9-2.6)	1.2 (1-1.5)	0.6 (0.5-0.8)	0.6 (0.5-0.8)
	ICC	41.6 (37.8-45.6)	40.1 (36.3-44.1)	26.9 (23.1-31)	16.2 (12.7-20.4)	16.1 (12.5-20.3)
	VE	0	6.1	48.5	72.9	73.2
Sao Tome and Principe	V_2	0.3 (0.2-0.6)	0.3 (0.1-0.5)	0.3 (0.1-0.6)	0.3 (0.1-0.6)	0.3 (0.1-0.7)
	ICC	9.1 (4.8-16.5)	7.1 (3.5-13.8)	8.2 (4.2-15.5)	7.2 (3.2-15.5)	8.1 (3.7-16.7)
	VE	0	23.9	10.3	22	12.2
Swaziland	V_2	0.2 (0.1-0.5)	0.2 (0.1-0.5)	0.2 (0.1-0.5)	0.3 (0.1-0.8)	0.3 (0.1-0.9)
	ICC	6.4 (2.9-13.8)	5.9 (2.4-13.7)	5.5 (2-14.1)	7.6 (2.6-20)	7.7 (2.6-20.9)
	VE	0	8.3	15.7	-20.3	-22.1
Zambia	V_2	0.5 (0.3-0.8)	0.5 (0.3-0.8)	0.3 (0.2-0.6)	0.3 (0.1-0.5)	0.3 (0.1-0.5)
	ICC	14 (9.5-20.2)	13.4 (9.1-19.2)	9.4 (5.9-14.6)	7.4 (4.2-12.9)	7.5 (4.2-13.2)
	VE	0	5.4	36.5	50.8	50

V_2 –Random variance at the community level, ICC –Intra-class correlation, VE –Variance explained. M0-M6 –Models 0 to 6, FA1 –Fully-adjusted model 1, FA2 –Fully-adjusted model 2. ICC and VE are reported as %.
